# Biological and substitute parents in Beaker period adult–child graves

**DOI:** 10.1038/s41598-023-45612-3

**Published:** 2023-10-31

**Authors:** Nicoletta Zedda, Katie Meheux, Jens Blöcher, Yoan Diekmann, Alexander V. Gorelik, Martin Kalle, Kevin Klein, Anna-Lena Titze, Laura Winkelbach, Elise Naish, Laurent Brou, François Valotteau, Foni Le Brun-Ricalens, Joachim Burger, Maxime Brami

**Affiliations:** 1 Palaeogenetics Group, Institute of Organismic and Molecular Evolution (iomE), Johannes Gutenberg University Mainz, Mainz, Germany; 2 Department of Environmental and Prevention Sciences, University of Ferrara, Ferrara, Italy; 3 Institute of Archaeology Library, LCCOS, University College London, London, UK; 4 Vor- Und Frühgeschichtliche Archäologie, Institut Für Altertumswissenschaften, Johannes Gutenberg University Mainz, Mainz, Germany; 5 The Culture Trust, Luton, UK; 6 Institut National de Recherches Archéologiques (INRA), Bertrange, Luxembourg

**Keywords:** Archaeology, Cultural evolution, Genomics

## Abstract

Joint inhumations of adults and children are an intriguing aspect of the shift from collective to single burial rites in third millennium BC Western Eurasia. Here, we revisit two exceptional Beaker period adult–child graves using ancient DNA: Altwies in Luxembourg and Dunstable Downs in Britain. Ancestry modelling and patterns of shared IBD segments between the individuals examined, and contemporary genomes from Central and Northwest Europe, highlight the continental connections of British Beakers. Although simultaneous burials may involve individuals with no social or biological ties, we present evidence that close blood relations played a role in shaping third millennium BC social systems and burial practices, for example a biological mother and her son buried together at Altwies. Extended family, such as a paternal aunt at Dunstable Downs, could also act as ‘substitute parents’ in the grave. Hypotheses are explored to explain such simultaneous inhumations. Whilst intercommunity violence, infectious disease and epidemics may be considered as explanations, they fail to account for both the specific, codified nature of this particular form of inhumation, and its pervasiveness, as evidenced by a representative sample of 131 adult–child graves from 88 sites across Eurasia, all dating to the third and second millennia BC.

## Introduction

This paper presents new evidence for the genetic relations of individuals from two double graves discovered in Altwies in 2000^1,2^ and in Dunstable Downs in 1887^3^, believed to date from the Beaker period. Three whole genomes at ~ 2X are reported here, as well as target enriched genomic data from one individual. New anthropological analysis and radiocarbon dates are also presented.

Today, the death of a child represents a traumatic event, invoking grief and bereavement. Children's burials are often private, family matters, with community engagement occurring only when tragedy or violence evoke a public response. Past reactions to child death were perhaps more complex and diverse, and in the archaeological record arguably find their clearest expression in surviving traces of funerary rites. Whilst we must be wary of applying modern, Western concepts of parenting and family structure to prehistoric communities, the close bond between child and adult, be they biological parent or social care-giver, appears to find expression through shared burials, perhaps when death overcame both at the same time. At the Mesolithic site of Vedbaek in Denmark, for instance, a newborn was buried with a juvenile woman on a swan’s wing^4^. Shared burials signal more than emotion; they may also serve, for example, to highlight family status lineage, or the place of children in the descent system.

The biomolecular evidence of close kin relations revealed by this study allows us to explore more fully a form of burial that has long intrigued archaeologists through the specific perspective of biological or family relationships. If the inhabitants can be shown to have been placed in the grave together, as intertwined, fully-fleshed bodies, we need to consider if they died naturally, within hours of each other, or suffered more sinister deaths. Interpretations of these burials as human sacrifices or premature burials were common amongst British antiquarians and archaeologists^3,5,6^. The German academic tradition postulated that young children rarely survived their mother in prehistoric times^7–10^; killing motherless children would have relieved matriarchal societies from the duties of child-care in the absence of marriage institutions^8^. Even today, shared burials are regarded as a proxy for massacres and ‘raids’^11,12^ or linked to infectious diseases and even major epidemics, such as the plague^13^.

## Background to the study

The practice of burying adults and children together in later European prehistory is known in German literature as ‘mother–child burials’ (‘Mutter und Kind Bestattungen’^10^). At the turn of the third millennium BC, societies predominantly buried their dead in single graves^14,15^, marking a widespread shift from collective to individual inhumation^16–19^. For such societies, shared inhumations represent an exception, hence the characterization of adult–child burials as “special” or “deviant burials” in archaeological literature^20^. Shared burials in which inhumations took place simultaneously must be distinguished from consecutive burials, such as those found in Unterhautzenthal, Austria^21^, or in Bell Beaker central Iberia, for example Camino del Molino, and La Atalayuela (La Rioja), where large deposits of human bones result from skeletons being pushed aside over time to make space for new inhumations^22–24^, and from intercutting graves, widely found in Beaker cemeteries.

Widespread changes in burial practices have been linked to the advent of pastoral populations from the Pontic-Caspian steppes, or at least their descendants, who admixed in various proportions with European Neolithic farmers and settled as far west as Britain c. 2,500 BC^25–29^. The discovery of first-generation immigrants to Wessex, such as the ‘Amesbury Archer’, who presumably spent his childhood in Continental Europe, around the western Alps^30,31^, has helped to rekindle interest in the so-called ‘Beaker folk’^32–34^. The movement of this new population into Northwest Europe has been portrayed as violent and disruptive, notably in popular studies of ancient genetics^35,36^, whilst monumental landscapes such as Stonehenge appear to have been radically transformed in the wake of their arrival^37^.

**Figure 1:**
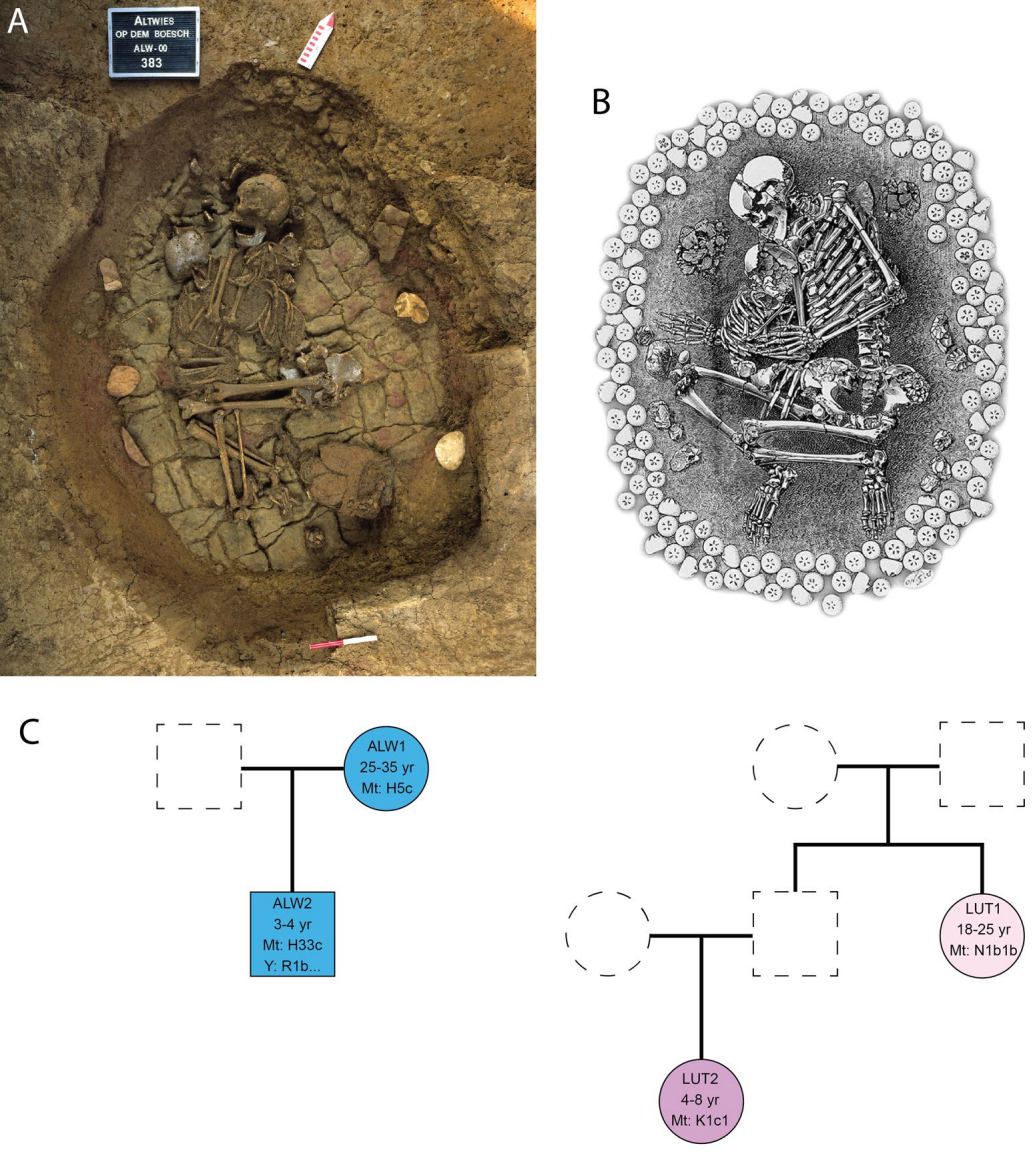
Adult–child graves sampled for this study, Bell Beaker period. (**A**) Altwies ‘‘Op dem Boesch’’, Luxembourg (photograph: Le Brun-Ricalens et al*.* 2011: Fig. 71^42^; *Institut National de Recherches Archéologiques*); (**B**) Dunstable Downs, Southern Bedfordshire, United Kingdom (etching: W.G. Smith 1894^3^); (**C**) inferred pedigrees for the individuals buried in Altwies (left) and Dunstable Downs (right). Key: circle: female; square: male. Colored shapes represent individuals with genetic data and dashed shapes, inferred individuals buried somewhere else.

Genetic analysis of two adult–child burials, from Altwies and Dunstable Downs, provides an ideal opportunity to explore the nature of shared burial practices in Beaker-period societies. These graves are incredibly similar in layout, to the extent that the same arrangement of stones can be observed on either side of the skeletons (Fig. 1)^2^. Were the child and the adult buried simultaneously? Were they biologically related, or was this a social relationship in death? If they were biologically related, what was the degree of relatedness between them? Few Beaker-period graves have produced genetic information about shared burials^27^. The phenomenon has received more attention in Central Europe, within the context of Late Neolithic to Early Bronze Age societies^15,20,38–41^.

## Results

### The adult–child burial of Altwies: archaeological and anthropological context

Two graves of the Bell Beaker culture were discovered in 2000, during rescue excavations at Altwies “Op dem Boesch”, Grand Duchy of Luxembourg, in advance of the construction of Highway A13 near the French border^2^. They were cut in the slope of a Jurassic plateau, c. 275 m in height, offering panoramic views of the valley of the Moselle river, a tributary of the Rhine^43^. While the plateau was densely populated in Early Neolithic times, no trace of occupation beside the two graves could be ascribed to the Bell Beaker period (Luxembourg Final Neolithic)^44,45^. The region between the Saar and the Moselle rivers has yielded over 100 Beaker-period habitation and funerary sites, including those at Sehndorf in Saarland (Germany) and Montenach in Moselle (France), both about 10 km away from Altwies; Beaker graves are usually found isolated or in groups of two to four, more rarely in small necropolises^46,47^.

New and previously published AMS dates from Altwies, based on human bones and charcoal samples, place the inhumations in the second half of the 3^rd^ millennium BC (Table 1). Grave 1 was probably that of a young adult male, accompanied by a retouched flake, the tip of a stone point and a flint strike-a-light^1^. This skeleton yielded no DNA due to the poor preservation and incomplete survival of the bones. The woman and child were buried c. 25 m further upslope, in an oval pit, Grave 2, c. 1.85 m in length and 1.45 m in width. Prior to burial, a fire was lit at the bottom of the pit, resulting in a cracked reddish floor and burned walls. The bodies were subsequently deposited alongside artefacts that showed no evidence of burning, including a stone ‘ring’, possibly made of fossilized shell, and a largely complete maritime-style Beaker^42^.

**Table 1:** Archaeological and genetic information for the individuals sampled. Mean sequencing depth is reported for shotgun whole genome sequencing data [w] and 1240 K capture regions [c]. For details on uniparental markers, see SI Table ‘Haplogrep results’, ‘Yleaf results’.

**ID**	Site	Country	^14^C age	Mean depth (X), w/c	Age at death	Molecular sex	Hapl mtDNA/# of reads	Hapl. Y/# of reads
ALW1	Altwies “Op dem Boesch”	Luxembourg	Beta-145714: 3680 ± 40 BP (2198–1947 calBC)	0.44/7.85	25–35	XX	H5c/4939	-
ALW2	Altwies “Op dem Boesch”	Luxembourg	MAMS-56230: 3656 ± 21 BP (2135–1950 calBC)	2.39/2.70	3–4	XY	H33c/20,269	R1b-PF6570 /243,222
LUT1	Dunstable Downs	UK	MAMS-58671: 3299 ± 32 BP (1666–1500 calBC); MAMS-61441: 3263 ± 27 BP (1613–1453 calBC)	2.24/2.51	18–25	XX	N1b1b/16,264	-
LUT2	Dunstable Downs	UK	MAMS-58672: 3021 ± 21 BP (1386–1201 calBC); MAMS-61442: 3182 ± 26 BP (1502–1417 calBC)	1.84/2.05	4–8	XX	K1c1/14,150	-

Dates calibrated in OxCal v4.4.4, using the IntCal20 calibration curve^48,49^.

The skeletons were seemingly placed and posed in the grave simultaneously—the head of the three to four-year-old child resting, for instance, in the right hand of the 25–35-year-old woman^2^. The woman’s body was placed in a flexed position on the right side, while the child was buried on the left side, the two individuals facing each other in death (Fig. 1A). Some anatomical articulations have been disturbed, suggesting that the grave was not backfilled, but left as an empty cavity, which may have been initially covered by a rigid frame, possibly a timber cap, and a small burial mound^2^. An examination of the bones failed to determine a cause of death for either individual.

### The ‘echinoid burial’ of Dunstable Downs: a 140-year old puzzle

The second burial under examination, the so-called ‘echinoid burial’, recovered from a round barrow on Dunstable Downs, Bedfordshire, is more problematic. In 1887, work to level the damaged barrow revealed human bones. Worthington George Smith (1835–1917), a local antiquary and naturalist, became involved in the retrieval of what he described as the crouched remains of a ‘girl’ and child discovered in a small, shallow grave in the east side of the barrow. Smith subsequently produced a drawing of the grave (Fig. 1B) for his book, *Man, the Primeval Savage*^3^.

The soft, fragmentary remains^50^, were ‘cleaned, dried, and then soaked […] in thin, hot (almost boiling) gelatine’, and dried again (^3^, 336). Once the bones were hard, they were fitted together with glue dissolved in spirit^3,50^. After Smith’s death, the remains passed to local naturalist Jannion Steele Elliot, who donated them to the Pritchard Memorial Museum at Bedford Modern School where the burial was displayed in the entrance hall^50,51^. Today, the remains are held at Luton Cultural Trust store. The use of such antiquarian materials always brings concerns about provenance and reliability, but Smith valued accuracy of representation and was well-respected within the nascent British archaeological community. The history of the remains post-excavation is well documented^50^.

The barrow, now destroyed, was located on the summit of Dunstable Downs and is designated barrow 8 of a dispersed cemetery of late Neolithic/early Bronze Age date focused on the seven barrows of the Five Knolls barrow cemetery. It lies 0.4 km south-east of Five Knolls^52,53^. The geology of the Downs is Late Cretaceous chalk, and the plateau is c. 244 m in height, providing panoramic views over the Vale of Aylesbury. The river Ouzel rises close by as a chalk stream. The barrow, originally 3 m high and 14 m in diameter, appears to have been multi-phase; Smith recorded that there was a large central grave, already completely robbed, whilst the grave examined here was one of ‘six or seven’ peripheral, secondary, burials, all located about 0.92 m from the surface. Of these other graves, two were empty, others contained fragmentary human remains, and a third contained a cremation and funerary urn^3,52^.

The woman was between 18 and 25 years of age; the child was four to eight years old^3,52^ (supplementary material SI 4). Smith depicted the woman lying on her left side, holding the child, with her head to the north (Fig. 1). Two broken pots were deposited near the woman’s head; a hammerstone, and white quartz pebble near her right hand. Elsewhere in the grave were two other hammerstones, two flint scrapers, and flint flakes. A flint arrowhead was lost during excavation^3,54^. Numerous fragments of aurochs were also found in the grave^55^. Dyer identified 45 fragments of undecorated early Bronze Age pottery, five beaker sherds with whipped cord and stabbed decoration, and eight ‘rustic’ beaker sherds from the excavations, but it is unclear whether these are from the grave or barrow^52^.

Perhaps the most remarkable of all grave finds were fossil echinoids (sea urchins), hence references to the grave as ‘the echinoid burial’. Initially, Smith reported 12 echinoids ‘surrounding the girl’, but ‘on extending the grave’, 91 were reportedly found and a further 200 from the entire barrow^3^. On balance, it is likely that the fossils came predominantly from the barrow, perhaps occurring naturally, or incorporated in the burial monument as ‘natural’ offerings^56^. In his drawing of the burial, Smith used the echinoids as a decorative border, which Dyer^52^ has claimed is unlikely to be accurate. However, such a placement of natural flint nodules and chalk lumps around a body is reported from burials in Yorkshire and Wessex, notably Barrow Clump, Wiltshire, where the crouched inhumation of a child was surrounded by a series of flint nodules (^57^, 105; 167).

New ^14^C dates obtained for the adult and child appear to be anomalously young (Table 1), considering the archaeological material associated with the burial, which is Bell Beaker (2500–1800 BC) in appearance and seems to share characteristics with Beaker graves in Wessex and Yorkshire. Garwood^58^ proposes that large multi-phase mounds, located in dominating landscape locations and containing many different age and gender categories, such as at Dunstable, can be dated to 2150–1800 BC. Given the use of animal-based products for bone preservation, contamination problems were suspected^59^ and the dates were repeated, at the CEZA Laboratory in Mannheim, using the inner part of the ear bone. Repeating the dating produced dates that are consistent with the adult and child being from the same period, but still about 500 years younger than expected under the assumption that Dunstable Downs and Altwies were contemporaneous (Table 1).

### Genetic relationship between the adult and the child

**Figure 2:**
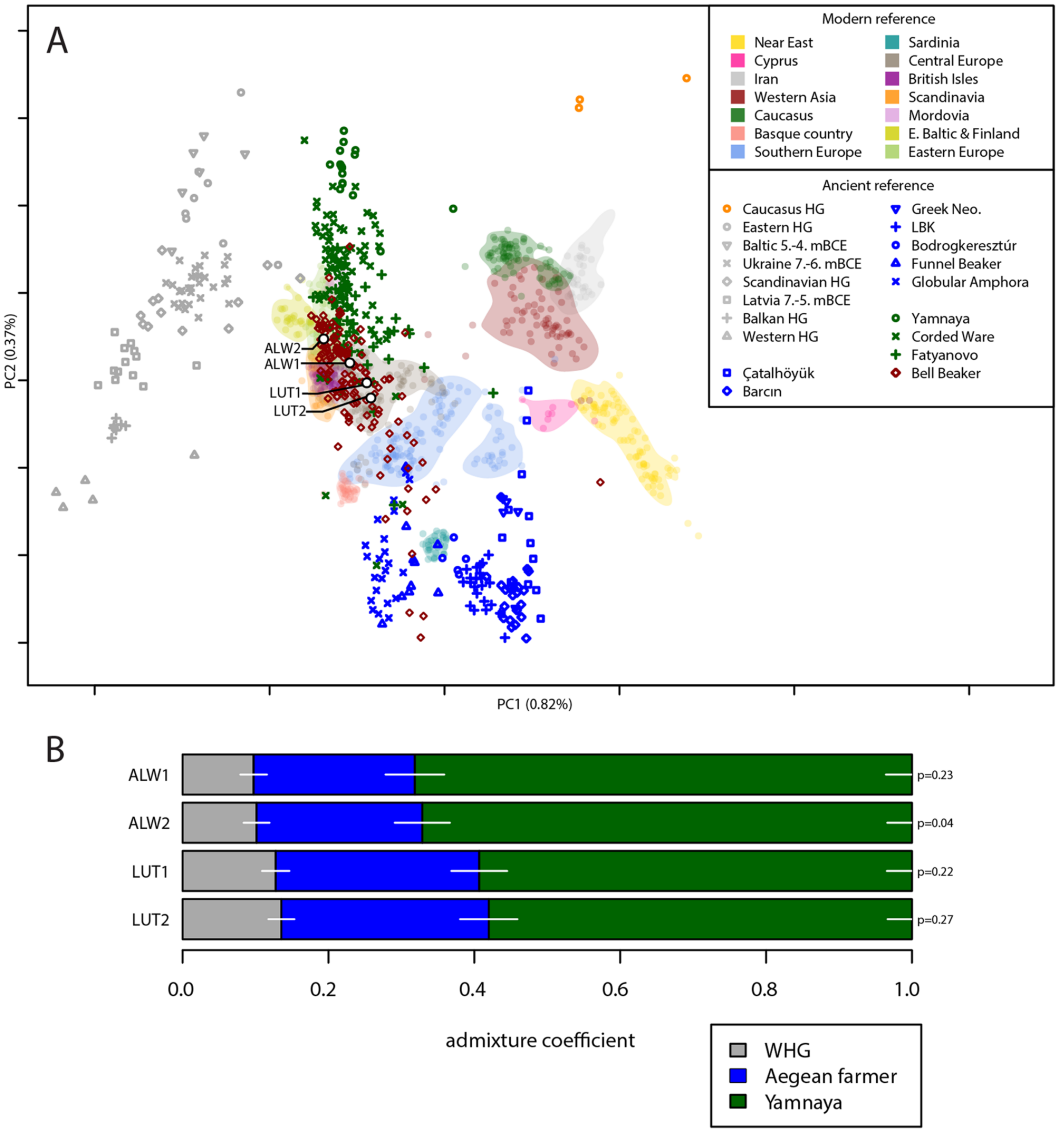
(**A**) PCA of modern and projected ancient West Eurasians and Southwest Asian individuals, highlighting the Altwies and Dunstable Downs individuals; ancient reference individuals are plotted according to the cultural phenomenon or subsistence basis to which they are traditionally assigned (legend in the top right corner); (**B**) Altwies and Dunstable Downs individuals modelled as mixtures of Western hunter-gatherer (WHG), Aegean–Anatolian farmer, and steppe-related (here represented by Yamnaya) ancestry components with *qpAdm.*

**Figure 3:**
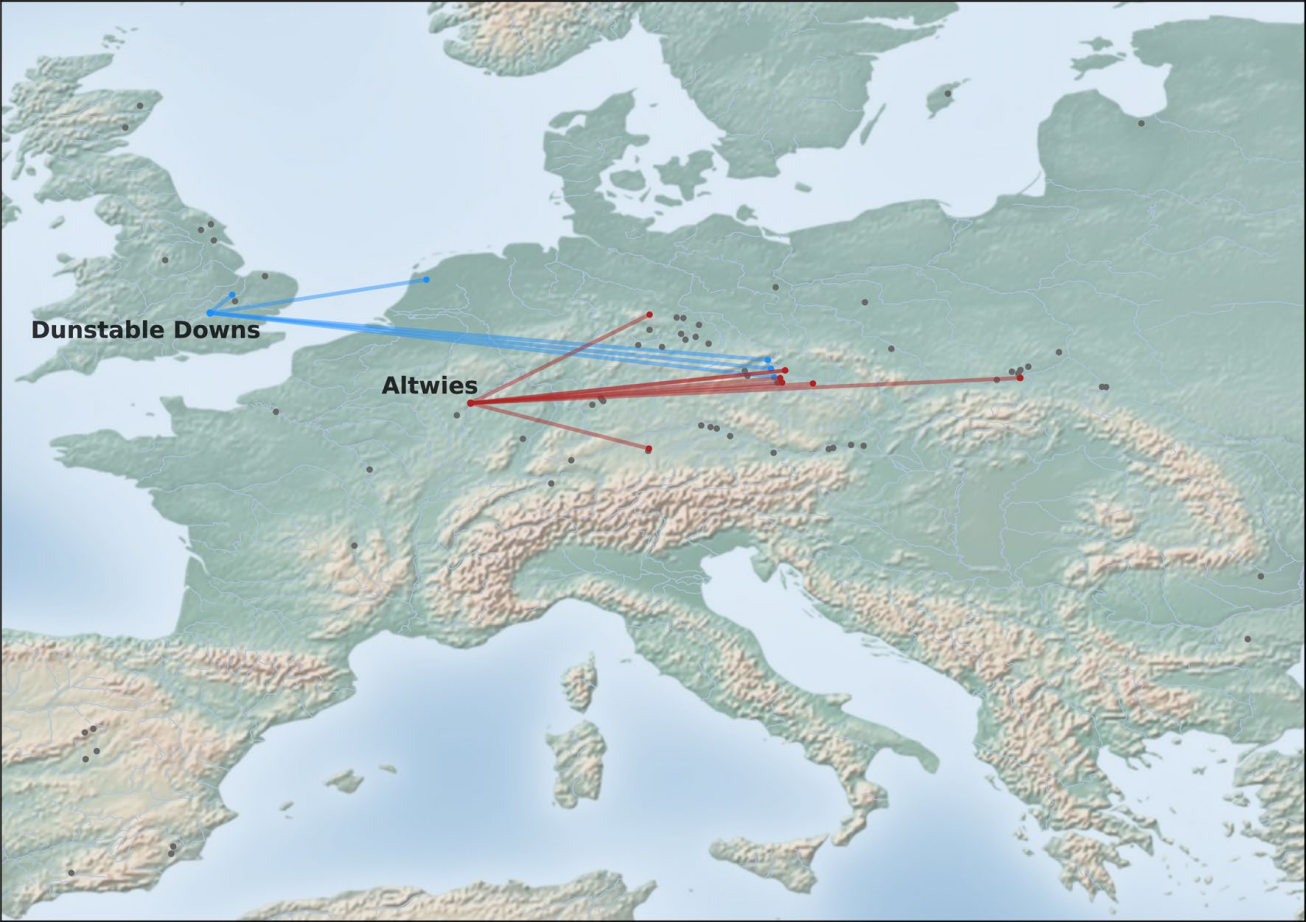
Location of Altwies “Op dem Boesch” (red dot) and Dunstable Downs (blue dot). Archaeological sites, in which individuals were found to share at least one IBD segment of ≥ 16 cM with our newly reported genomes are shown in the same color. Sharing patterns are further highlighted by a vector line. Relevant third- and second-millennium BC adult–child graves are represented by grey dots. Adult–child graves from the Eurasian steppe belt are not depicted here. For a full list of burials and descriptions, see Table 2 and Supplementary Materials, Fig. S1. Matplotlib Basemap Toolkit 1.3.8 and Python 3.11 were used to produce the map. As map background, a display shaded relief image (from http://www.shadedrelief.com) was used.

**Figure 4:**
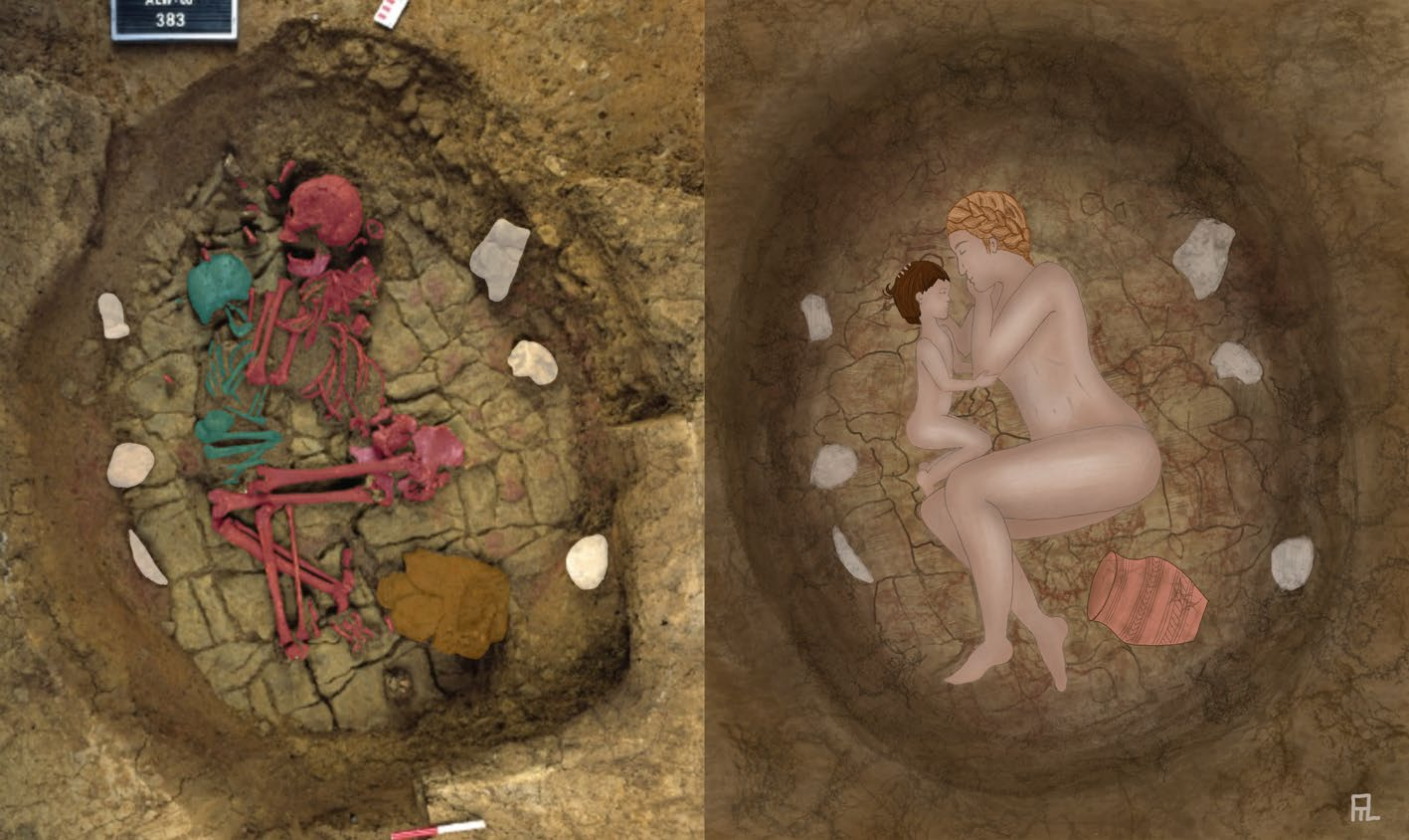
The grave of Altwies, left: the bones of the mother and child highlighted (photograph: Le Brun-Ricalens et al*.* 2011: Fig. 71^42^; *Institut National de Recherches Archéologiques*); right: hypothetical reconstruction of the grave based on phenotypic traits partly inferred from the ancient genomes.

The adult and child were biologically related in both cases. We were able to sequence whole genomes with low contamination estimates (MT < 1%, autosomal < 3%) and establish the degree of relatedness between the adult and child at Altwies and Dunstable Downs using the pairwise mismatch rate and KIN (see method section ‘Genetic Relatedness’). The four individuals are relatively similar on a PCA of modern and ancient Eurasians and show high levels (ALW1 68.2%; ALW2 67.2%; LUT1 59.4%; LUT2 58%) of steppe-related ancestry in a three-population qpAdm model (Fig. 2). The amount of steppe ancestry is consistent with the Beaker expansion in Northwest Europe^27,31^, a conclusion emphasised by identity by descent (IBD) segments of ≥ 16 cM shared between the four individuals and contemporary genomes from England, the Netherlands, Germany, Bohemia and Poland, ascribed to Bell Beaker and contemporary sites (Fig. 3; supplementary materials, Fig. S2). A particular hotspot emerges in Bohemia, in sites attributed to the Corded Ware, the Bell Beaker and the Únětice cultures^27,60^. Although intriguing, we caution against overinterpreting these results, without a deeper understanding of the underlying demographic history of these individuals. Furthermore, the possibility of a sampling bias, based on the availability of reference genomes cannot be ruled out. The absence of long runs of homozygosity excludes recent inbreeding. While the two graves look similar archaeologically, we found no evidence at genomic level that the two pairs of individuals were ever in contact. Pigmentation phenotypes for hair, skin, and eyes (SI Table ‘HIrisPlex results’) were produced for all four individuals, and used for the reconstruction in Fig. 4.

Our analyses indicate that the child at Altwies, who was genetically male, was buried alongside his biological mother (Fig. 1). Both individuals shared the same mitochondrial haplogroup, H (Table 1). A difference at two SNPs was observed (12,127 G- > A, 16188C- > T), which is likely the result of post mortem damage and differences in coverage. The boy’s Y haplogroup, R1b, is at high frequency in Bell Beaker males^29^. The orientation of the woman at Altwies may be regarded as anomalous. In Continental European Beaker communities, women and girls were placed on their right side, with the head at the south, whereas men and boys were buried on their left side, with their head at the north—both sexes thus facing the eastern direction^61^. Conversely, in Altwies, the woman was placed with her head to the north, facing west. The orientation of the grave thus apparently aligned with the sex of the child.

At Dunstable Downs, both the child and the adult were identified as genetically female. The two individuals were second degree-related on the paternal side (Fig. 1). They belonged respectively to the K1c and N1b mitochondrial haplogroups and therefore had different mothers. Given the pairwise mismatch rates and the age of the adult compared to the child, we can exclude half-sibling and grandmother-granddaughter relationships. The adult is likely to be a paternal aunt. According to Smith, the head of the crouching female child was to the north^3^. It is not clear if the orientation of the grave was intended for the child or the adult. The orientation of Bell Beaker burials is generally less strict in the south of England, with females lying either on the left side with the head to the north, or on the right side with the head to the east^62^.

## Discussion

### A widespread phenomenon in third millennium BC Western Eurasia

In order to shed further light on the burials, we looked for archaeological parallels beyond Altwies and Dunstable Downs. Adult–child burials cut across virtually all major cultural horizons in third millennium BC Western Eurasia, occurring in Bell Beaker, Corded Ware, and Yamnaya cemeteries, as indicated by the representational list of graves in Table 2 and in the Supplementary Materials, and were surprisingly common, even in societies that predominantly practised single inhumation. Child burials of all periods are rare in the archaeological record, not in the least with regard to special burials, grave forms, and artefact associations^58^. Many children died young and did not receive formal burial. Established archaeological studies of children and childhood emphasise the need to assess children as active cultural and social agents and participants in their societies, in both life and death^63^.The cultural significance of children in the early Bronze Age is uncertain and the treatment of their physical remains varied greatly^58^. Double burials containing both adults and children therefore offer us rare glimpses into their contemporary social and cultural importance alongside adults.

**Table 2:** List of third and second millennium BC adult–child burials from Eurasia. For full descriptions, plates and references, see Supplementary Materials (numbers refer to site IDs in Fig. S1). Biological age groups after Martin and Saller (1957)^93^.

ID	Site	Sex and age
Bell Beaker and related		
1	Achenheim	? inf. II + ♀ adu
4	Altwies “Op dem Boesch”	♂ inf. I + ♀ adu
6	Augy “Ferme de Champagne”	? inf. + ♂ juv./adu
14	Broomend	? inf. + ♂ ? adu
15	Caminos de las Yeseras	? inf. I + ? adu. + ? adu. + ? adu
		♀ inf. I + ♀ adu
16	Camino del Molino	Collective burial (30% subadult)
21	Dunstable Downs	♀ inf. I/II + ♀ adu
25	Ganton Wold	? inf. + ♀ adu
28	Hanging Grimston Group	? inf. I/II + ? adu
29	Hatrize	? inf. I + ♂ adu. + ? adu
30	Hay Top, Little Longstone	? inf. + ? adu
31	Humanejos	? inf. + ♂ adu. + ♂ adu. + ? adu. + ? adu
32	Irlbach	? inf. I + ♀ juv./adu
39	La Almoloya	♀ neo. + ♀ adu
		♀ neo. + ♀ adu
40	La Atalayuela	Collective burial (20% subadult)
41	La Bastida	♂ neo. + ♂ adu
42	La Salmedina	? inf. + ♂ adu
		? inf. + ♀ adu
45	Łęki Małe	♀ inf. II/juv. + ♂ adu
46	Léry, Les Petits Près 2	? foet. + ? foet. + ♀ adu
47	Mains of Melgund	? inf. II + ♂ adu
51	Mortimer’s Barrow 4	? inf. II + ♂ mat./sen
57	Pont-du-Château/Chazal	? inf. + ♀ adu
68	Szarbia Zwierzyniecka	? inf. I + ♂ sen
74	Trumpington	♀ juv. + ♂ juv./adu
77	Valle de las Higueras	? inf. I + ? inf. II + ♀ adu
84	Willerby	? inf. I + ♀ sen
Pitted-Ware		
78	Västerbjers	? inf. + ♀ adu
		? inf. + ♂ juv./adu
Globular Amphora		
35	Koszyce	♂ inf. I + ♂ inf. I + ♂ inf. I + ♀ inf. II + ♀ juv. + ♂ juv. + ♂ juv./adu. + ♀ adu. + ♀ adu. + ♀ adu. + ♀ adu. + ♂ adu. + ♀ mat. + ♂ mat. + ♂ mat
Corded Ware and related		
3	Althausen	♀? inf. I/II + ♂? Inf. II + ♂ adu. + ♀ mat
5	Atting-Ringkam	? inf. + ? adu
7	Auleben	? inf. I + ♂ adu
11	Blšany	? neo. + ♀ adu
17	Chłopice	♀ inf. II + ♀ inf. II/juv
18	Chrášťany	♀? inf. II/juv. + ♂ juv./adu
22	Eulau	? inf. I + ♀ adu
		? inf. I + ? inf. I + ♂ adu
		? neo. + ♀ inf. I + ♂ inf. II + ♀ adu
		♂ inf. I + ♂ inf. II + ♀ adu./mat. + ♂ mat
24	Gabułtów	? inf. + ? inf. I + ♂ adu
36	Künzing	? inf. I + ♀ mat./sen
43	Lauda-Königshofen	? neo. + ♂? inf. I + ♀ adu
		? foet. + ♀ adu./mat
		♀? inf. I + ♀ adu
		? neo. + ♀? inf. I + ? inf. II/juv. + ♀ juv
		? neo. + ? inf. II + ♀ adu./mat
		? inf. I + ? inf. II + ? juv
50	Marschwitz / Marszowice	? inf. + ? inf. + ♀ adu
53	Oechlitz	? inf. I + ? inf. I/II + ? inf. II + ♂ inf. II/juv. + ? adu./mat
62	Selgas	? inf. I + ♀ mat
65	Spreitenbach-Moosweg	? neo. + ? inf. II/juv. + ♂ juv. + ♀ adu. + ♀ adu. + ♀ adu. + ♂ adu. + ♂ adu. + ♂ adu. + ♂ adu. + ♀ mat. + ♂ mat
66	Stetten a. D. Donau	? neo. + ♀ adu
67	Święte	? inf. II + ♂ mat. + ♀ mat
69	Tauberbischofsheim-Dittigheim	? juv. + ♀ adu./mat
		? inf. + ? inf. + ♀ adu
		? inf. I + ? inf. II + inf. II/juv
		? inf. I + ? inf. II + ♂ juv. + ♀ adu. + ♀ adu./mat. + ♂ adu./mat
70	Tauberbischofsheim-Impfingen	? neo. + ? inf. II/juv. + ♀ adu. + ♂ adu
		♂ inf. II + ♂ adu. + ♂ mat./sen
72	Tiefbrunn	♀ inf. + ♂ adu. + ♂ sen
73	Trebnitz	? inf. + ♀ adu
80	Vikletice	♂? juv. + ♀? adu
		? inf. II + ♀? adu
		? inf. I + ♀? adu./mat
		? inf. I + ♂? adu
		? inf. I + ♂? adu./mat
		♀inf. II/juv. + ♂? juv
81	Wangenheim	? inf. + ♀ adu
82	Węgrzce	? inf. I + ♂ adu./mat
83	Weimar	? inf. + ? adu
85	Wojciechowice	? inf. II + ♀? juv./adu
87	Żerniki Górne	? inf. + ♂ mat
		? inf. II + ♂ mat
		♀ juv. + ♀ adu
		? inf. I + ♀ juv./adu
		? inf. I + ♀ mat
		? inf. I + ♀ mat
		? inf. II + ♂ juv./adu
		? inf. II + ♀ adu
88	Złota, st. 59	? inf. I + ? inf. II + ? inf. II + ♀ adu./mat
Fatyanovo, Balanovo, Volosovo		
12	Bolshenevskii	♂ inf. + ♀ juv. + ♀ adu. + ♂ adu
38	Kuz’mino	? neo. + ♂ adu
9	Balanovo	? inf. I + ♀ adu
86	Yazykovo I	? inf. + ♀ adu
Yamnaya and related		
19	Chudomir	? inf. + ? adu
48	Mamai-Hora	? inf. + ♀ mat
49	Mar’yanskii	? inf. + ♂ adu
52	Nerushai	? inf. + ♀ adu
55	Pervomaiskii	? inf. + ? adu
60	Salhir	? inf. + ♀ adu
64	Smeeni	? inf./juv. + ♂ adu./mat
Afanasievo, Okunevo and related		
2	Afanas’eva Gora	♂ juv. + ♀ mat./sen
		? inf. II + ♂ sen
10	Bike I	? inf. I + ♀ adu
20	Chernovaya VIII	♀ inf. II + ♀ adu. + ♂ adu
33	Khuurai Govi 1	? inf. + ♂ adu
54	Okunev Ulus	? + ♀ adu
59	Sal’dyar-1	? inf. I/II + ♀ adu./mat
71	Tas Khazaa	? inf. + ♀ adu. + ♀ adu. + ♂ adu
		? inf. + ♀ adu. + ♂ sen
79	Verkhnii Askiz I	? inf. II + ♂? adu./mat
Katakombnaya		
8	Baburskii	? inf. + ? adu
26	Grigor’evka	? inf. I + ? inf. I/II + ? inf. II + ♂ adu
37	Kut	? inf. + ? inf. + ♀ adu
55	Pervomaiskii	? inf. + ? adu. + ? adu
Únětice and related		
13	Brehna	? inf. I + ♂ mat
23	Franzhausen I	? foet. + ♀ mat
		? foet + ♀ adu
		♂ inf. II + ♂ adu./mat
		? foet + ♀ adu
27	Haid	? inf. + ? adu
34	Königsbrunn-Obere Kreuzstraße	? inf. + ♀ adu
44	Leau	♀ inf. II/juv. + ♀ juv./adu. + ? juv./adu. + ♂ adu
56	Plötzkau	♂? inf. I + ♂ juv./adu
		? inf. I + ? inf. I + ♂ juv. + ♂ juv./adu. + ♀ adu
58	Röcken	? inf. II + ? inf. II + ♀ adu. + ♂ adu
61	Schleinbach	? inf. I + inf. II + ? inf. II + ♂ adu
63	Serbitz	? inf. II + ? inf. II + ♂ adu./mat. + ♂ mat
75	Unterhautzenthal	? neo. + ♀ inf. II/juv
		? inf. I + inf. I + ♀ adu./mat
		? inf. I + ♀ juv./adu
		? neo. + ♀ mat./sen
		? inf. I + ♀ mat./sen
		? neo. + ♀ juv./adu. + ♂ adu./mat
76	Unterwölbling	? inf. I + ? inf. I + ♂ sen
		? inf. I + ? inf. II

Children's graves are generally under-represented in Beaker-period cemeteries from Northern France and Britain^64–66^. Some can be described as mere imitations of adult graves, with miniature vessels, weapons and items of personal adornment^57,67^. Others were intended for children as well as adults. Shared burials from France, such as those of Achenheim and Léry, consisted of children and babies placed in front or back-to-back with a crouched woman^68,69^. A three to five-year old child was discovered by the side of an adult man and the cremated remains of another adult in the rectangular pit grave or burial chamber of Hatrize in the Grand Est region of France^70^. The close relationship between the skeletal remains of the adult and the child implies that both individuals were interred at the same time. Evidence from Early Bronze Age Britain indicates that children, and indeed women, seldom went to the grave alone. Here, multiple burials were the most usual form of burial for both adults and subadults^71,72^. Between 30 and 40% of child burials from Scotland, Yorkshire, and Wiltshire were buried with an adult, with a bias towards burial with females^57,58^. At the Beaker cemetery of Craig Tara Holiday Park, Ayrshire, Scotland, for example, every child was buried in a multiple grave containing an adult^73^.

Looking further afield, shared burials were remarkably frequent in some Corded Ware cemeteries of Central Europe. Up to 44% of graves in two cemeteries from the Tauber Valley, Southern Germany, contained more than one individual^11^. Intriguingly, shared burials are mainly found outside the area of distribution of regular child graves in Corded Ware Central Europe, suggesting two burial traditions instead of one^14^. At Eulau, Oechlitz, Lauda-Königshöfen, and Tauberbischofsheim, small clusters of two to five individuals, including adults and children, were buried together in simple pits^14,74–76^. Ancient DNA analyses indicate in this instance close genetic relatedness between some of the adults and children^38–40,77^. The Únětice culture, which succeeds and partly overlaps Corded Ware and Bell Beaker traditions in Central Europe, provides many examples of shared burials, some of which have since produced biomolecular evidence for parent–child inhumations^15,20^.

The origins of this practice remain unclear. The fact that shared burials in Altwies and Dunstable Downs were associated with individuals with high levels of steppe-related ancestry may indicate the spread of a new burial practice among steppe pastoralists and their descendants, representing innovations in cultural, ritual, and possibly symbolic attitudes and beliefs^16,17^. Early examples of adult–child burials in pit graves under burial mounds, or kurgans, can be observed in the Yamnaya core area in the Pontic-Caspian steppes^78^. Double and multiple inhumations of adults and children occur frequently here. The dead are often arranged in theatrical displays of personal affection, such as hugs, kisses, or eye contact, that resemble those observed in Corded Ware and Bell Beaker cemeteries^10,14^. Furthermore, subadults may have been preferentially buried with an adult in Yamnaya graves^78^. Looking further East, Central Asian Afanasievo graves, which are linked to the expansion of steppe populations to South Siberia and Mongolia^79^, featured similar shared burials^80^.

Transformative attitudes to family identity and social status may have been central to the adoption of these new practices, affecting individual bonds within families, which could have contained both biologically and non-biologically related members, and communities. Increased diversity in burial rites, particularly double and triple burials with infants, and the appearance of child burials with elaborate grave goods suggests that at least in death, children had become increasingly important^57,81^. Both women and children may have been regarded as of relational importance in death as in life, symbols of fertility or status, or responsible for creating and cementing transgenerational links between and within communities, even linking past and present^71^. This link may also explain the frequent occurrence of the disarticulated remains of children found buried with adults in the early Bronze Age. This practice has been interpreted by Garwood^58^ as evidence of children as ‘grave goods’, but equally, the remains of children could have been stored until the death of a socially or ritually appropriate family or community member permitted burial.

### Biological and substitute parents

Our study provides the first genetic evidence that Bell Beaker communities in Northwest Europe buried children with their biological mothers and other close biological relatives, apparently in accordance to a specific rite practised throughout the wider ‘Beaker’ world, and indeed beyond. Conversely, Olalde et al*.* found no close genetic relationship between co-buried individuals at the Bell Beaker necropolis of Camino de las Yeseras, in San Fernando de Henares, Spain^27^. A young adult woman, who had a typical ancestry of the Iberian Peninsula, was buried atop the skeleton of an infant girl in an artificial cave. The infant was neither her daughter nor a close biological kin, but had a comparatively high amount of steppe ancestry^27,82,83^. We may speculate that kinship practices were different in the southern domain of the Bell Beaker culture, where collective burial traditions in megalithic tombs and artificial caves continued uninterrupted^23^. Alternatively, close ties of kinship, and perhaps circumstances of death, needed to be articulated through a specific performative ritual language of side-by-side body posture and rite.

While genetic evidence remains scarce, a variety of kinship patterns are reflected in third millennium BC shared burials. That the adult and child at Dunstable Downs were second-degree related on the paternal side might hint at a patrilineal descent system, in which the paternal aunt could play the role of substitute parent or primary care-giver. At Altwies, the grave’s orientation matched the sex of the boy, not that of his biological mother. Central European Corded Ware graves have previously yielded evidence for ‘nuclear’ families—referring here to biological parents and their offspring^38^. At Eulau, a single pit grave included the remains of four intertwined skeletons, placed in pairs facing each other; the father and one son were placed with the head to the West, the mother and another son with the head to the North^38^. The orientation of the children’s skeletons deviated from the Central European Corded Ware tradition, emphasizing the relationship with their parents instead of their biological sex^38^.

Some of the children in Corded Ware graves were buried with adults who were not their biological parents, making it hard to generalize observations; not all kinship relationships would have been biological. At Eulau, two children, who were probably siblings, were placed by the side of a woman, who had a different mitochondrial haplogroup^38^. She may have been a paternally-related aunt, a stepmother, or a completely unrelated individual; the exchange of foster children as part of kinship systems, or social and political networks has been proposed^84^**.** The Althausen grave in Baden-Württemberg belonged to a ‘patchwork’ or blended family. It consisted of a man and a woman facing each other, with two children placed between them, who were not related to each other^40^. Burial with a family member or care-giver may have been the preferred rite for children, but in their absence, perhaps any adult may have served, the creation of proxy families or relationships perhaps seen as necessary for the safe passage of the child through death.

### The interpretation of parent–child burials

While the causes of death could not be established for the Altwies and Dunstable Downs individuals, the sheer number of actual and potential parent–child burials identified in the third millennium BC (Table 2) raises further questions about the interpretation of this burial form. Did the buried individuals die naturally or were they killed? Birth accidents can be ruled out in all but a few examples, such as the grave of an adult woman and a neonate at the Corded Ware site of Stetten a. d. Donau in Germany^85^. Natural child deaths should not be discounted; 40% of children in prehistoric societies may have died before the age of five^57^. However, the simultaneous inhumation of children and adults, who appear to be close relatives or care-givers, is more difficult to explain. Epidemics, for instance *Yersinia pestis*, common across Eurasia during the Bronze Age^86^, could theoretically explain the death of multiple individuals at close intervals, but data remain lacking for third millennium BC shared burials.

Interpretations of the mother–child bond in shared burials might also evoke suspicions of violence, sacrifice, or even honour killings. That women and children were frequently the victims of violence in late Neolithic/Early Bronze Age Europe is undisputed. At Eulau, Germany, one of the adult women buried with a child suffered an arrow wound, described as ‘unambiguously fatal’^11^. The killing of children, for instance by blunt force to the skull, has been documented at several Early Bronze Age sites in Europe, including Schleinbach in Lower Austria, Cezavy Hill in the Czech Republic, and Nord-Trøndelag in Norway^87–90^.

Smith^3^ believed that the child at Dunstable Downs had been ‘buried alive with the mother’. Such willingness to accept the idea of human sacrifice was widespread amongst early prehistorians and indeed, can still be found in literature today. In the process of forming ideas for their new discipline, early prehistorians relied heavily both on Classical writers, such as Herodotus, who described the alleged strangling of retinue members and servants in Scythian barrows (Herodotus, Histories IV, c. 71), and contemporary ethnography, leading them to conceptualise the early Bronze Age peoples of Europe as ‘savages’ or barbarians and adopt sinister or violent explanations. Today, such notions seem fantastical. However, we should not discount the possibility that ritual killing and/or human sacrifice was practised in later prehistoric Europe^91^.

We should take care not to perceive all adult–child burials as the same. Non-kinship patterns such as relative age, sex, manner of death and social standing, could be just as important as kinship in determining how to handle the death of community members. The burials examined in this article were socially significant events and did not lack care or elaboration; they were furnished as regular graves with beakers and status items. Preparation for the inhumation of the mother and son at Altwies likely spanned two days; the burial pit was cleansed by fire prior to the deposition of the bodies and the artefacts^2^. Given such care, ‘ad hoc’ interpretations, such as burial following enemy raids, are problematic. Do all shared burials represent the aftermath of raids, when joint burial was offered to victims by survivors? At Eulau, five of the 13 skeletons placed in the four shared burials displayed perimortem injuries, including defence wounds^11^. These explanations, evoking actual crime scenes, fail to reflect the repetitive and codified nature of shared burial practices in third millennium BC Western Eurasia.

For Garwood^58^ funerary displays described social and political biographies in individualised if idealised and aesthetically consistent ways. Perhaps the key to understanding the specific examples of ‘parent–child’ burials described here lies not in their potential cause of death, but in the specific, nuanced configuration of the bodies in the grave; two individuals buried in each other’s arms (Fig. 4). Turek^92^ has observed that one of the most highly symbolic elements within Corded Ware burial rites is the position of the buried person’s arms. This was not related to distinctions between sex and age groups, nor did it appear to relate to grave goods. This positioning continued into the Beaker period and may relate to a social category/identity as yet unclear. The body of a woman, lying as though sleeping, clasping a child in her arms, obviously had a specific meaning to early Bronze Age peoples, a meaning retained across thousands of miles and amongst many diverse and fluid contemporary funerary practices. Whatever it was, it represented something powerful and emotive.

## Material and methods

### Sample preparation

All laboratory analyses were carried out at the dedicated ancient DNA facilities of the Palaeogenetics Group, Institute of Organismic and Molecular Evolution (iomE) at the Johannes Gutenberg University, Mainz, according to strict ancient DNA protocols to prevent contamination with modern DNA as well as cross-contamination between samples^94–96^, including decontamination of workspace, lab-ware and samples, and processing of negative controls during all steps (sample pulverisation, DNA extraction, library preparation and PCR reactions). All the steps before PCR amplification were conducted in the dedicated ancient DNA facility physically separate from post-PCR areas.

One petrous bone, part of the temporal bone, was collected for each of the four individuals. The dense core of the petrous bone contains the highest amount of endogenous DNA, and is therefore the optimal sampling area of ancient skeletons^97^.

The petrous bones were decontaminated under ultraviolet light (254 nm) from 2 sides for 45 min per side, then cleaned with a sandblasting machine (P-G 400, Harnisch + Rieth, Winterbach, Germany) to remove the outer bone surface and soil. The densest, non-porous, inner part of the petrous bone was then isolated and cut in small cubes using a disk saw (Marathon N7, SMT), which were irradiated again with ultraviolet light (254 nm) from two sides for 45 min per side and pulverized using a milling machine (MM200, Retsch). Blank milling controls containing hydroxyapatite were processed in parallel with the samples to control contamination.

### DNA extraction

The modified Yang et al*.* (1998) protocol^98–100^, with some additional modifications^101,102^ described below, was followed for DNA extraction.

The bone powder, 0.10–0.15 g per sample, underwent a pre-lysis by being incubated with 1 ml of EDTA (0.5 M, pH 8) at room temperature for 10 min prior to extraction. The supernatant was discarded after the solution was centrifuged at 10,000 rpm to pellet the powder.

Using 1.8 ml of an extraction buffer made of EDTA (950 µl, 0.5 M, pH8), Tris–HCl (20 µl, 1 M, pH8), N-Lauroylsarcosine (17 µl, 5%), and Proteinase K (13 µl; 20 mg/ml), lysis was carried out on rocking shakers (1400 rpm) at 37 °C for 48 h.

Following the 48-h incubation period, the samples were centrifuged for 10 min at 10,000 rpm to separate the supernatant, which was then transferred to an Amicon Filter (Amicon Ultra-4 30 kDA, 15 ml) and centrifuged for 10 min at 2500 rpm. The DNA was then washed twice with 3 ml 1X Tris–EDTA followed by centrifugation at 2500 rpm for 20 min and removing the flow-through in between. After washing, the extract was concentrated to 100 μl and then purified using the MinElute PCR Purification Kit as directed by the manufacturer, but incubating for 5 min during the elution with 44 μl elution buffer (preheated to 65 °C).

During DNA extraction, blank controls were processed and used in all subsequent analysis stages.

### Library preparation

Double-indexed Illumina libraries were created according to the protocol by Kircher et al*.* (2012)^103^ with slight modifications. The damage patterns of the DNA fragments were used to show that there was no modern DNA contamination. For whole genome sequencing the extracts were treated with USER™ enzyme prior to library preparation: 5 μl of USER™ enzyme was added to 16.25 μl of DNA extract and the mixture was incubated for 30 min at 37 °C.

The blunt-end repair was performed using the NEBNext End Repair Module (New England Biolabs, Ipswich, Massachusetts, United States): 20 µl of DNA extract (or 16.25 µl + 5 µl of USER™ for non-screening libraries) were mixed with NEBNext End Repair Reaction Buffer (10X, 7 µl), NEBNext End Repair Enzyme Mix (3.5 µl) and nuclease-free water (39.5 µl for screening or 38.25 µl for non-screening libraries; for a final reaction volume of 70 µl) and incubated for 15 min at 25 °C followed by 5 min at 12 °C.

In the adapter ligation step, hybridized adapters P5 and P7 (IDT, Leuven, Belgium)^104^ were used at a concentration of 0.75 µM.

To add unique and sample-specific index pairs to the library molecules, 3 µl of the fill-in product (total volume: 40 µl) were amplified using the AccuPrime™ Pfx SuperMix (20 µl) in one PCR parallel (final reaction volume: 25 µl; final primer concentration: 200 nM). Double indexing was done following Kircher et al*.* (2012)^103^, but with index sequences from the NexteraXT index Kit v2 (Illumina; barcode length 8 bp).

The PCR was carried out in 10–14 cycles; the temperature profile used for the PCR complied with the manufacturer's guidelines, but used an annealing temperature of 60 °C, extending for 30 s during each cycle, and carrying out a final elongation step for 5 min.

Purification during library preparation was conducted using the MinElute PCR Purification Kit (Qiagen, Hilden, Germany), while amplified libraries were purified with the MSB® Spin PCRapace kit (Invitek, Stratec Molecular, Berlin, Germany). Library concentrations were quantified by Qubit Fluorometric quantitation (dsDNA HS assay) and measurement on the Agilent 2100 Bioanalyzer System (High Sensitivity DNA Analysis) was used to estimate fragment length distributions of the libraries.

To confirm the success of the library preparation and to monitor contamination, blank controls, as well as positive controls (nonsense hybrids) of known concentration, were processed during every library step, including PCR amplification. A quantitative analysis of the blank controls revealed no significant contamination in any laboratory stage (pulverisation, DNA extraction, library preparation and amplification). A summary of all labwork and sequencing is reported in SI Table.

### Whole-genome sequencing

All four samples were firstly screened for their endogenous DNA preservation using shallow shotgun sequencing. Screening runs were performed on an Illumina MiSeq™ platform at StarSEQ GmbH (Mainz, Germany) in single-end runs with 50 bp read length. After adapter trimming and quality check^103^, reads were aligned to the human reference genome GRCh37/hg19 using BWA aln^105^, duplicates were removed and reads were filtered for a minimum length of 30 bp using the MarkDuplicates function from the Picard tools package (picardtools, http://broadinstitute.github.io/picard). The percentage of endogenous DNA was calculated as the ratio of unique aligned reads to the reference genome against the total number of reads after quality filtering. One sample had very low endogenous content (ALW1) at 1.4%, the other three between 20 and 50% (SI Table ‘General Stats’). Post-mortem damage patterns in aligned, length-filtered sequence reads were obtained with the software package MapDamage 2.0^106^ for all the libraries to evaluate sample authenticity.

For deeper whole genome sequencing, 2–3 DNA extracts and 3–9 libraries of each sample were prepared (as already described above). The libraries were amplified in up to 12 PCR parallels to increase the complexity of the libraries.

The number of cycles for the PCR reactions varied between samples (10–14 cycles) as it was adjusted to the presumed quantity of DNA fragments in the library. After amplification, all parallels of the same library were purified together and quantified as described above. For sequencing, libraries were pooled according to their concentrations measured on Qubit® and taking into account the endogenous DNA content as estimated by MiSeq sequencing and subsequently purified with Agencourt® AMPure® XP beads.

The samples were sequenced on an Illumina NovaSeq6000 (SE, 100 cycles) at the Next Generation Sequencing Platform at the University of Berne, Switzerland.

### Target enrichment

Screening showed that one sample (ALW1) had very low endogenous content. This sample was target enriched using the Twist Bioscience ancient DNA Target Enrichment reagent, following the protocol of Rohland et al*.* (2022)^107^ with the modifications described below.

Two independent capture reactions were performed for sample ALW1, the first with two independent double-indexed libraries originating from the same DNA extract, and the second using additional libraries from a second DNA extraction resulting in a total of four libraries (see SI Table for details).

The double-indexed libraries were pooled and concentrated to obtain 1500 ng (instead of 1 library of 1000 ng) that are then reconstituted in 7 µl of universal blockers and 5 µl blocker solution. Following the indication of Rohland et al. (2002)^107^, we mixed 1 µl of Twist Ancient DNA probes (Twist custom probe panel number: TE-94002772, Part Number: 104298) and 0.167 µl of the MT probes (Part Number: 102040 with 5 µl of hybridization mix. The probes were heated for 2 min at 95 °C and subsequently cooled for 5 min at 4 °C. After heating libraries and blockers for 5 min at 95 °C, both solutions were left at room temperature for 5 min. The probes mix (6.167 µl) was added to the 12 µl library and blocker, mixed, overlaid with 30 µl hybridization enhancer and incubated at 62 °C in a thermal cycler for 16 h. We used 300 µl streptavidin beads and bound the targets for 30 min at room temperature on a shaker (600 rpm). The beads were then washed four times with two different wash buffers; three were stringent washes at 49 °C. Only 50% of the bead slurry (the rest was frozen and stored) was then amplified with 25 µl Equinox Library Amp Mix in 12 cycles with 2.5 µl of the provided primers (ILMN). The amplified capture products were then purified with 1.8 × DNA purification beads and eluted in 32 µl Elution buffer.

The two capture reactions were then sequenced on an Illumina NovaSeq6000 (SE, 100 cycles) at the Next Generation Sequencing Platform at the University of Berne, Switzerland, and afterwards, the data were merged.

### Bioinformatics pipeline

Residual adapters were removed with trimmomatic 0.36^108^ and sequences shorter than 30 bp were discarded prior to aligning the reads to the human reference genome (GRCh37/hg19) with bwa mem^109^. During conversion to BAM format using samtools^110^ only reads with a mapping quality ≥ 30 (-q 30) were kept. PCR duplicates were removed from the alignment with sambamba markdup^111^ and the remaining reads were realigned around known SNPs and InDels using GATK 3.6^112^. Potential contamination was assessed based on reads aligning to the mitochondrial genome using contamMix 1.0.9^113^ and for autosomal reads using ContamLD^114^, together with ANGSD^115^ for X-chromosomal reads in ALW2. Since the ANGSD estimates suggested contamination levels below 1%, we used the ContamLD estimates for ALW2 for correcting the estimates for the remaining genomes. Genetic sexing of each individual was done following Skoglund et al*.* (2013)^116^.

#### Variant call and phasing/imputation

Variant detection was done with ATLAS^117^. After estimating deamination patterns for sequenced libraries separately, the sequencing error was estimated jointly based on a set of sites, known to be ultra-conserved among multiple mammalian species, following the description at the bitbucket repository:

https://bitbucket.org/wegmannlab/atlas/wiki/Home.

The “majorityBase” function was used for sites overlapping the 1240 K capture regions described in Mathieson et al*.* (2015)^118^ and the mitochondrial genome to generate pseudo-haploid genotype calls. Diploid calls for a set of sites determined to be bi-allelic and present in at least two individuals in the 1000 Genomes data-set^119^ were produced with the “MLE” call-function. To further minimise potential artefacts introduced by PMD, we ignored the first and last 2 bp of each read during calling.

Phasing/imputation of the genomes was performed by running glimpse 1^120^ following the provided instructions and using default parameters with the 1000 genomes data as reference^119^.

#### Uniparental markers

MtDNA- and Y-chromosomal haplotypes were determined with HaploGrep3^121^, and Y-leaf^122^ with the ISOGG 2018 tree (International Society of Genetic Genealogy) respectively.

### Patterns of variation

#### PCA, qpAdm

Explorative genomic similarity analysis was performed by PCA using LASER v.2.04^123^ following Hofmanová et al.^102^ projecting BAM files onto a reference space of modern Eurasian populations^124^. Modern reference populations used: Southern European (Italian North/South, Sicilian, Spanish/- North, Canary Islander, Maltese, Greek), Basque, Sardinian, Cypriot, Central European (Albanian, Bulgarian, Romanian, Hungarian, Croatian, Czech, German, French), Eastern European (Russian, Ukrainian, Belarussian, Polish, Sorb), Mordovian, Eastern Baltic and Finnish (Estonian, Lithuanian, Finnish), British Isles (English, Orcadian, Scottish, Irish/-Ulster, Shetlander), Scandinavian (Icelandic, Norwegian), Caucasian (Georgian, North Ossetian, Abkhasian, Chechen, Adygei, Lezgin, Kumyk, Balkar), West Asian (Turkish, Armenian), Iranian/-Bandari, and Near Eastern (Palestinian, Druze, Jordanian). Ancient reference individuals are given in SI Table ‘PCA references’ (annotation from the Allen Ancient DNA Resource^125^).

*f*_*4*_ admixture proportions were computed with *qpAdm* from ADMIXTOOLS^126^ using default parameters and on pseudohaploid calls at the positions overlapping with the human origin/Illumina capture. Outgroups for *qpAdm* were individuals with labels Han.SDG, Karitiana.SDG, Mbuti.SDG, and Papuan.SDG^127^ and ancient genomes Russia_MA1_HG.SG, Ethiopia_4500BP_published.SG, Belgium_UP_GoyetQ116_1_published, Russia_Ust_Ishim.DG, Spain_ElMiron, all retrieved from the Allen Ancient DNA Resource v50.0^125^.

#### IBD and ROH analyses

Screening for runs of homozygosity was done for each individual using hapROH^128^. Shared haploid IBD segments between our newly reported genomes and a set of previously published individuals (for a detailed list see Supplementary Table 7) were determined with ancIBD^129^ focussing on sites in the 1240 K capture array^118^.

### Phenotypic inference

The HIrisPlex-S webtool^130^ was used to predict pigmentation phenotypes for hair, skin, and eyes for each sample. Results were used for the reconstruction in Fig. 4.

### Genetic relatedness

Genetic relatedness was assessed by computing pairwise-relatedness coefficient *r* for autosomal and X-chromosomal SNPs following Fowler et al. (2022)^131^ with a set of contemporary genomes from previously published studies (see SI Table for further details) in addition to running KIN^132^. Directionality of the inferred relatedness was resolved by comparing autosomal and X-chromosomal *r*-values, in addition to the assessment of IBD patterns estimated by KIN.

### Anthropological analyses

Detailed anthropological analysis of the Beaker-period skeletons recovered in Altwies took place in 2000 and is reported in Toussaint et al.^2^. Anthropological examination of the two individuals from Dunstable downs was carried out to detect the presence of any pathological or traumatic lesions that could have led to their death. The age at death was estimated for the subadult through the analysis of dental eruption and the development of various skeletal parts and the degree of fusion of ossification centres^133,134^. The adults’ age-at-death was estimated using the degenerative changes due to ageing in the skeleton, in particular dental wear and ectocranial suture obliteration^135–137^. Paleopathological examination and trauma detection was carried out through macroscopical examination of the skeletons. The results of the analysis are reported in the Supplementary Materials.

### Radiocarbon dating

New radiocarbon dates were obtained for three of the individuals sampled (ALW2, LUT1, LUT2). In addition, a distal phalanx of the left thumb of the adult at Altwies (ALW1) was dated in 2000 to 2198–1947 calBC at 2σ (Beta-145714: 3680 ± 40 BP^1^). For new samples, the analyses were conducted at the Curt-Engelhorn-Zentrum Archäometrie gGmbH (Mannheim, Germany). The collagen was extracted from fragments of the petrous bones used for aDNA analysis. All dates were calibrated in OxCal v4.4.4, using the IntCal20 calibration curve^48,49^. The date obtained for the child at Altwies, 2135–1950 calBC (ALW2: MAMS-56230: 3656 ± 21 BP) is consistent with the date of the mother (ALW1: R_Combine value of 2135–1955 calBC at 2σ, X2-Test: df = 1 T = 0.3(5% 3.8)).

For Dunstable Downs, the adult and child initially produced dates that were internally inconsistent at 2σ, despite the second-degree kinship relationship between them. The dates were respectively 1666–1500 calBC (LUT1: MAMS-58671: 3299 ± 32 BP) and 1386–1201 calBC (LUT2: MAMS-58672: 3021 ± 21 BP) (Table 1). Contamination issues were suspected, due to the use by Smith of gelatin and ‘shellac dissolved in spirit’ to glue together the bones (^55^, 319). Shellac is a bioadhesive polymer, a solution of melted lac, the resinous excretion of the Lac insect (*Coccus* or *Carteria lacc*a), formerly used extensively in archaeological conservation^138^. Re-dating of the samples in Mannheim, removing the bone surface before pretreatment, appears to have eliminated the age discrepancy between the two individuals. The adult and child are now respectively dated to 1613–1453 calBC (LUT1: MAMS-61441: 3263 ± 27 BP) and 1502–1417 calBC (LUT2: MAMS-61442: 3182 ± 26 BP) (Table 1). It is unclear if the gelatin only contaminated the surface of bones or also soaked into the material, but in any case the treatment could have produced artificially younger dates^59^. The dates should therefore be treated with caution. In addition, MAMS-61441 shows a very low collagen preservation (0.4%).

### Supplementary Information

Supplementary Information 1.

Supplementary Information 2.

## Data Availability

Sequencing data are available in FASTQ and BAM format at the European Nucleotide Archive under the accession number PRJEB65118. VCFs and raw outputs from ancIBD used to make Fig. 3 are available at: https://irods-web.zdv.uni-mainz.de/irods-rest/rest/fileContents/zdv/project/palaeogenetik/IBDres.tar.gz?ticket=0O2nNMMnBbyN4KL.
