# Supplementary material for: Biological and substitute parents in Beaker period adult–child graves
Source: Sci Rep. 2023 Oct 31;13:18765. doi: 10.1038/s41598-023-45612-3 (PMC10618162; doi:10.1038/s41598-023-45612-3)
Supplement: Supplementary file 2 — Supplementary Information 2. [file 41598_2023_45612_MOESM2_ESM.pdf]

Supplementary Materials for

Biological and substitute parents in Beaker period adult-child graves

Authors

Nicoletta Zedda*[1,2], Katie Meheux*[3], Jens Blöcher[1], Yoan Diekmann[1], Alexander V. Gorelik[4], Martin Kalle[4], Kevin Klein[4], Anna-Lena Titze[1], Laura Winkelbach[1], Elise Naish[5], Laurent Brou[6], François Valotteau[6], Foni Le Brun-Ricalens[6], Joachim Burger[1], Maxime Brami*[1]

Affiliation

[1]Palaeogenetics Group, Institute of Organismic and Molecular Evolution (iomE), Johannes Gutenberg University Mainz, Mainz, Germany.

[2]University of Ferrara, Department of Environment and Prevention Sciences, Ferrara, Italy.

[3]Institute of Archaeology, University College London, United Kingdom.

[4]Vor- und frühgeschichtliche Archäologie, Institut für Altertumswissenschaften, Johannes Gutenberg University Mainz, Mainz, Germany.

[5]The Culture Trust, Luton, United Kingdom.

[6]Institut National de Recherches Archéologiques (INRA), Bertrange, Luxembourg.

*These authors contributed equally.

Correspondence to Maxime Brami (mbrami@uni-mainz.de)

List of contents

SI 1. Map of Eurasia (adult-child burials) 2

SI 2. List of shared burials with adults and children 4

SI 3. Map of Eurasia (IBD sharing patterns) 23

SI 4. Anthropology of Dunstable Downs 24

Supplementary References 27

Supplementary Information 1. Map of Eurasia (adult-child burials)

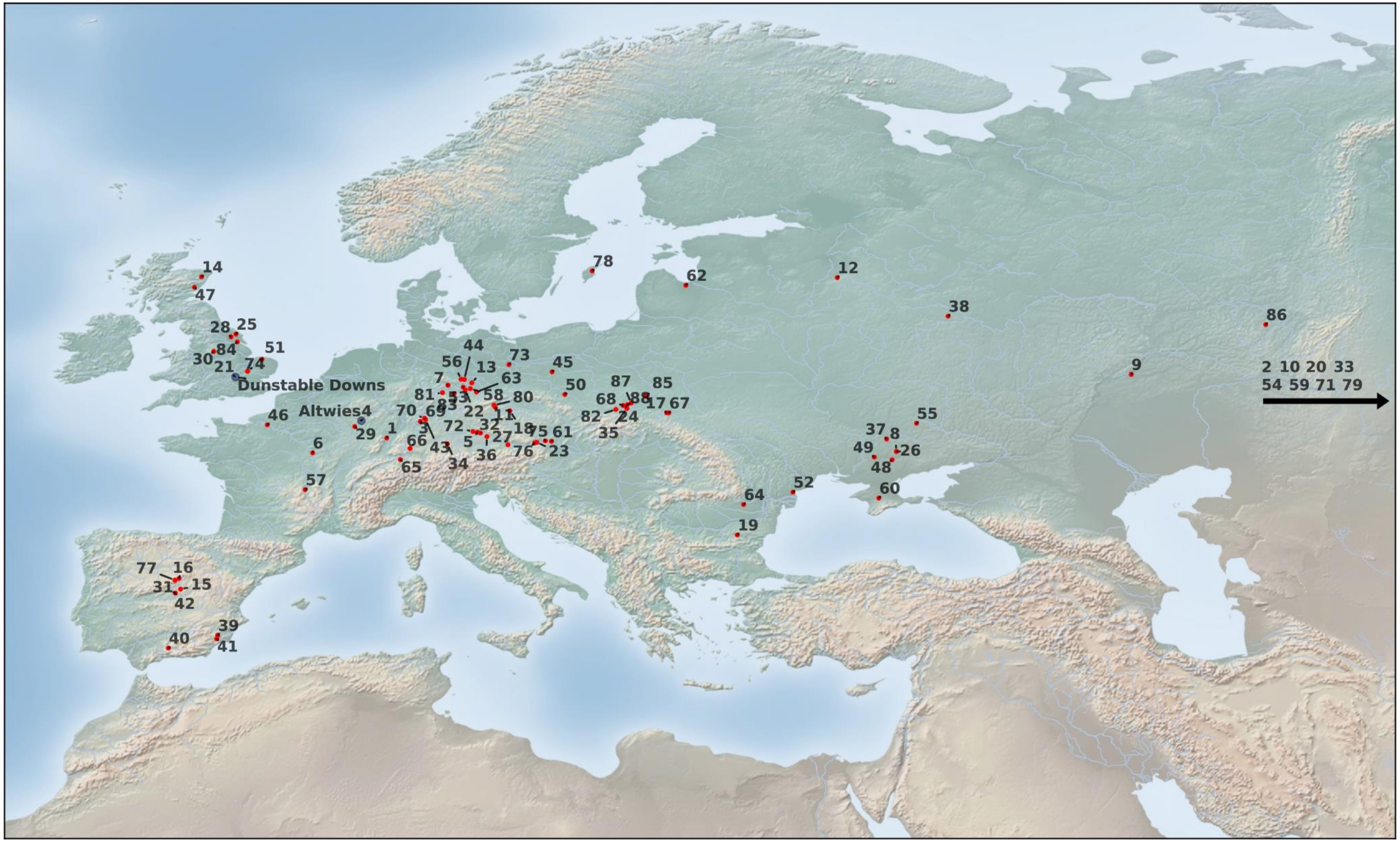

Figure S1. Map of Eurasia showing the location of relevant third- and second-millennium BC adult-child burials. Matplotlib Basemap Toolkit 1.3.8 and Python 3.11 were used to produce the map. As map background, a display shaded relief image (from http://www.shadedrelief.com) was used. Legend: 1. Achenheim, Alsace; 2. Afanas'eva Gora; 3. Althausen; 4. Altwies "Op dem Boesch"; 5. Atting-Ringkam; 6. Augy "Ferme de Champagne"; 7. Auleben; 8. Baburskii; 9. Balanovo; 10. Bike I; 11. Blšany; 12. Bolshenevskii; 13. Brehna; 14. Broomend; 15. Caminos de las Yeseras; 16. Camino del Molino; 17. Chłopice; 18. Chrášťany; 19. Chudomir; 20. Chernovaya VIII; 21. Dunstable Downs; 22. Eulau; 23. Franzhausen; 24. Gabułtów; 25. Ganton Wold; 26. Grigor'evka; 27. Haid; 28. Hanging Grimston Group; 29. Hatrize; 30. Hay Top, Little Longstone; 31. Humanejos; 32. Irlbach; 33. Khuurai Govi 1; 34. Königsbrunn-Obere Kreuzstraße; 35. Koszyce; 36. Künzing; 37. Kut; 38. Kuz'mino; 39. La Almoloya; 40. La Atalayuela; 41. La Bastida; 42. La Salmedina; 43. Lauda-Königshofen; 44. Leau; 45. Łęki Małe; 46. Léry, Les Petits Près 2; 47. Mains of Melgund; 48. Mamai-Hora; 49. Mar'yanskii; 50. Marschwitz; 51. Mortimer's Barrow 4, Painsthorpe Wold; 52. Nerushai; 53. Oechlitz; 54. Okunev Ulus; 55. Pervomaiskii; 56. Plötzkau; 57. Pont-du-Château/Chazal; 58. Röcken; 59. Sal'dyar-1; 60. Salhir; 61. Schleinbach; 62. Selgas; 63. Serbitz; 64. Smeeni; 65. Spreitenbach; 66. Stetten a. D. Donau; 67. Święte; 68. Szarbia Zwierzyniecka; 69. Tauberbischofsheim-Dittigheim; 70. Tauberbischofsheim-Impfingen; 71. Tas Khazaa; 72. Tiefbrunn; 73. Trebnitz; 74. Trumpington; 75. Unterhautzenthal; 76. Unterwölbing; 77. Valle de las Higueras; 78. Västerbjers; 79. Verkhnii Askiz I; 80. Vikletice; 81. Wangenheim; 82. Węgrzce; 83. Weimar; 84. Willerby; 85. Wojciechowice; 86. Yazykovo I; 87. Żerniki Górne; 88. Złota.

.

Supplementary Information 2. List of shared burials with adults and children

Representative sample of 131 adult-child graves from 88 third and second millennia BC sites, for which information is available.
Key: country codes: AUT - Austria; CHE - Switzerland; CZE - Czechia; DEU - Germany; ESP - Spain; FRA - France; GBR - United Kingdom of Great Britain and Northern Ireland; LAT - Latvia; LUX - Luxembourg; MNG - Mongolia; POL - Poland; ROU - Romania; RUS - Russian Federation; SWE - Sweden; UKR - Ukraine.
Key: culture: AFA- Afanasievo; BAL - Balanovo; BBC - Bell Beaker; CWC - Corded Ware; EBA - Early Bronze Age; FAT - Fatyanovo; GAC - Globular Amphora Culture; KAT - Katakombnaya; OKU - Okunevo; PWC - Pitted-Ware; UNE - Únětice; VOL - Volosovo; YAM - Yamnaya.
Dates calibrated in OxCal v4.4.4, using the IntCal20 calibration curve [1,2].

**Site ID**	**Site**	**Country code**	**Barrow, Grave No**	**Culture**	**Date (calBC, 2σ)**	**Children**	**Adults**	**Body position, grave goods**	**Note, incl. degree of relatedness (as reported)**
4	**Altwies "Op dem Boesch"** [3,4]	LUX	Grave 2	BBC	2135-1955	Male (3-4 yr)	Female (25-35 yr)	Adult lying in flexed position on the right side, cradling a child, maritime beaker, stone ring	1st degree (mother-son), this study
21	**Dunstable Downs** [5,6]	GBR	Barrow 8, "echinoid burial"	BBC? EBA?	1666-1417	Female (4-8 yr)	Female (25 yr)	Adult lying in flexed position on the right side, cradling a child, 2 beakers, 2 stone axes, 6 scrapers, broken sharpening stone, flint flakes, 3 globular stone mauls, arrowhead	2nd degree (paternal aunt-niece), this study
1	**Achenheim** [7]	FRA		BBC		Indet. (8-10 yr)	Female	Adult and child buried back to back, 2 beakers, spindle whorl, bone tools	
2	**Afanas'eva Gora** [8,9]	RUS	Grave 26	AFA		Male (15 yr)	Female (40-60 yr)	Adult in supine position with flexed legs. Adolescent to the right of the adult, in supine position with flexed legs, 2 ceramic vessels, ochre, copper pieces, bracelet	

Site ID	Site	Country code	Barrow, Grave No	Culture	Date (calBC, 2σ)	Children	Adults	Body position, grave goods	Note, incl. degree of relatedness (as reported)
2	**Afanas'eva Gora** [8,9]	RUS	Grave 27	AFA		Indet. (13-14 yr)	Male (> 60 yr)	Adult in supine position with flexed legs. Adolescent to the right of the adult, in supine position with flexed legs, 2 ceramic vessels, 2 arrowheads	
3	**Althausen** [10,11]	DEU	Grave 29	CWC	2569-2462	Probably female (6-7 yr); probably male (9-11 yr)	Male (25-30); female (40-50 yr);	The two adults face each other. Two children are placed between them. The woman is in flexed position on the left side, cradling the boy. The adult male is in flexed position on the right side, holding the girl in his arms. No grave goods	"Patchwork family", not a father, a mother and two biological children according to mtDNA analysis (see [11], based on DNA from [12])
5	**Atting-Ringkam** [13,14]	DEU		CWC	2835-2476	Indet.	Indet.	Adult and child in an oval pit. The adult is in flexed position on the left side. The child is under the adult's right elbow behind him/her. Beaker, flint sickle, faunal remains	
6	**Augy "Ferme de Champagne"** [15,16]	FRA		BBC		Indet.	Male (18-25 yr)	3 beakers, 3 arrowheads, blade, scraper	
7	**Auleben** [14,17]	DEU	Barrow 2, grave 1	CWC	2848-2465	Indet. (Infans 1)	Male	Adult in flexed position on right side, child in flexed position on left side behind the adult at the feet, amphora, cord-decorated cup. Both under a small burial mound	
8	**Baburskii** [18]	UKR	Group 1, Kurgan 1, Grave 19	KAT		Indet.	Indet.	Adult in supine position. Child lying right of the adult, in flexed position on the right side, stone battleaxe, 2 bone pendants, charcoal, ochre	
9	**Balanovo** [19]	RUS	Grave 33	BAL		Indet. (5-6 yr)	Female (30-35 yr)	Adult in flexed position on the left side. Child to the left of the adult, disturbed position, ceramic vessel, copper/bronze decoration, snails, miniature wheels	Disturbed

Site ID	Site	Country code	Barrow, Grave No	Culture	Date (calBC, 2σ)	Children	Adults	Body position, grave goods	Note, incl. degree of relatedness (as reported)
10	**Bike I** [20]	RUS	Kurgan 35	AFA		Indet. (5-6 yr)	Female	Adult in supine position with flexed legs. Child to the right of the adult, in supine position with flexed legs, ochre	
11	**Blšany** [21,22]	CZE		CWC	c. 2650-2400 (as reported)	Neonate	Female	Adult in flexed position with head towards E, shell disc amulet, 2 hair-rings, 1 ceramic vessel, several beads, 6 animal teeth	
12	**Bolshenevskii** [23]	RUS	Kurgan 4	FAT		Male (inf.); female (juv.)	Female, male	Adult male in flexed position on the left side. Adolescent female behind adult male, in flexed position on the right side. Child in flexed position on the left side. Faunal remains incl. pig bones, perforated bear fang, shaft axe, flint knife, 2 Dreissena shells	Four individuals buried with a dog, signs of violence
13	**Brehna** [24]	DEU		UNE		Indet. (4 yr)	Male (40-50 yr)	Skeletons deposited in the remains of an oven, ceramic sherds, multiple salt pans	
14	**Broomend, Inverurie, Aberdeen** [25]	GBR	Stone cist grave	BBC		Indet.	Indet., possibly male	2 beakers, horn ladle, 2 flint flakes, possible remains of hide covering	
15	**Caminos de las Yeseras** [26]	ESP	Funerary area 21	BBC		Indet (5 yr)	3 indet. adults	16 beakers	
15	**Caminos de las Yeseras** [26]	ESP	Funerary area 2, artificial cave 1	BBC	2461-1702	Female (1-5 yr)	Female (20-30 yr)	Child at the far end of the artificial cave, directly covered by the skeleton of the woman, the latter buried in supine position with flexed legs, 2 regular-size and 1 miniature beakers	'Unrelated' [27]
16	**Camino del Molino** [28,29]	ESP		BBC				Collective burial; Around 30% of inhumed individuals subadult. No clear relation between subadult and adult individuals.	

Site ID	Site	Country code	Barrow, Grave No	Culture	Date (calBC, 2σ)	Children	Adults	Body position, grave goods	Note, incl. degree of relatedness (as reported)
17	**Chłopice** [30]	POL	Grave 11	CWC	2617-2351	Female (11-12 yr); female (14-15 yr)	-	Two subadults in flexed position on the left side in a niche construction. Younger individual resting on the knees of the older one, facing away from her, 2 beakers, bone awl, stone flake, 5 pendants of pierced animal teeth	2st degree relatives, same mtDNA haplogroup H2a2b [30]
18	**Chrášťany** [31]	CZE		CWC		Female? (14-16 yr)	Male (young); indet.	Three individuals in a burial pit. The adult and child buried in antipodal position, the adult male in flexed position on the right side, the subadult on the left side facing the opposite direction, various grave goods including pottery	
19	**Chudomir** [32]	BUL	Feature 5	YAM?		Indet.	Adult	Adult in supine position with flexed legs. Child to the left of the adult, in supine position with flexed legs, 3 ceramic vessels	
20	**Chernovaya VIII** [33]	RUS	Kurgan 5, Grave 10	OKU		Female (7-9 yr)	Female (20-25 yr); male (20-35 yr)	Individuals placed in flexed supine position with head toward W. Subadult individual placed on the chest of the female individual.	
22	**Eulau** [34–36]	DEU	Grave 90	CWC	2574-2350	Indet. (4-5 yr).	Female (25-35 yr)	Adult in flexed position on the left side, facing the child, animal tooth pendant	Arrow wound (adult) [35]
22	**Eulau** [34–36]	DEU	Grave 93	CWC	2862-2502	Indet. (4-5 yr); indet. (4.5-5.5 yr)	Male (25-40 yr)	Adult in flexed position on the right side, facing a child, another child buried beneath the adult, axe blade, pricker	Perimortem injuries (adult) [35]
22	**Eulau** [34–36]	DEU	Grave 98	CWC	2659-2476	Indet. (0.5-1 yr); female (4-5 yr); male (7-9 yr);	Female (30-38 yr)	Adult lying on the left side, facing a child, 2 children buried behind the woman, facing opposite directions, silex blade, axe blade, silex flake, pricker	Possible 1st degree (siblings); 'unrelated' (adult-children) [34], cranial trauma (adult) [35]

Site ID	Site	Country code	Barrow, Grave No	Culture	Date (calBC, 2σ)	Children	Adults	Body position, grave goods	Note, incl. degree of relatedness (as reported)
22	**Eulau** [34–36]	DEU	Grave 99	CWC	2840-2497	Male (4-5 yr); male (8-9 yr)	Female (35-50 yr); male (40-60 yr)	2 adult-child pairs in flexed position, facing opposite directions, 2 silex blades, axe blade, bone prickle/spur	1st degree relatives (mother-son, father-son, siblings) [34], cranial trauma (child) [35]
23	**Franzhausen I** [37–39]	AUT	Grave 139	UNE		Foetus	Female (40-60 yr)	Adult in flexed position on the right side	
23	**Franzhausen I** [37–39]	AUT	Grave 309	UNE		Foetus	Female (20-25)	Adult in flexed position on the right side	
23	**Franzhausen I** [37–39]	AUT	Grave 588	UNE		Male (10-12 yr)	Male (35-50 yr)	Adult and child in flexed position on the left side	
23	**Franzhausen I** [37–39]	AUT	Grave 941	UNE		Foetus	Female (30-40 yr)	Adult in flexed position on the right side	
24	**Gabułtów** [40]	POL	Grave 2	CWC		Indet. (infans); indet. (infans I)	Male	Niche grave	
25	**Ganton Wold, Yorkshire** [41]	GBR	Barrow 3	BBC?		Indet.	Female	Cremated bone and earlier disarticulated remains, Beaker and arrowhead?	
26	**Grigor'evka** [42]	UKR	Kurgan 3, Grave 6	KAT		Indet. (2 yr); indet. (5-7 yr); indet. (12-14 yr)	Male (30-35 yr)	Adult in flexed position on the left side, children to the left of the adult, infant in flexed position on the right side, child in flexed position on the left side, adolescent in flexed position on the left side, ochre	

Site ID	Site	Country code	Barrow, Grave No	Culture	Date (calBC, 2σ)	Children	Adults	Body position, grave goods	Note, incl. degree of relatedness (as reported)
27	**Haid** [43]	AUT	Grave 78A-B	UNE		Infant	Indet.	Adult in flexed position on the right side, embracing the infant, in flexed position on the right side	
28	**Hanging Grimston Group, Yorkshire** [44]	GBR	Barrow 12	BBC?		Indet. (6-8 yr)	Indet.	Flint-flaked knife, small heap of burnt bone	
29	**Hatrize, Meurthe-et-Moselle** [45]	FRA	Grave 247	BBC		Indet. (3-5 yr)	Male (30+ yr); indet./cremation (25+ yr)	Adult facing the child, buried in opposite direction with flexed legs, cremated remains of a 2nd adult in an organic container, 3 vases, arrowhead	Timber chamber?
30	**Hay Top, Little Longstone** [46,47]	GBR		BBC?		Infant	Indet.		
31	**Humanejos** [48]	ESP		BBC		Indet (15 yr)	2 adult men, 2 indet. adults	13 beakers, 2 wristguards, 4 Palmela points, tanged dagger and 3 gold plaques	
32	**Irlbach** [49]	DEU	Grave 2	BBC		Indet (infant I)	Female (young)	Adult flexed on right side, 1 plate/bowl	
33	**Khuurai Govi 1 / Kurgak gövi** [50–52]	MNG	Grave 1	AFA	3011-2473	Indet.	Male	Adult in supine position with flexed legs. Child to the right of the adult, in supine position with flexed legs, copper knife, copper awl, bone arrowhead, chipped stone tools, worked bone piece, animal tooth pendant, organic implement, faunal remains, incl. bones of ovicaprids	Female relative of child buried in nearby grave (Kurgak gövi 2) [53]
34	**Königsbrunn-Obere Kreuzstraße** [12,54]	DEU	Grave 33 (feature 8.9)	EBA		Indet.	Female		Possibly 'unrelated' (different mtDNA haplogroups) [12,55]

Site ID	Site	Country code	Barrow, Grave No	Culture	Date (calBC, 2σ)	Children	Adults	Body position, grave goods	Note, incl. degree of relatedness (as reported)
35	**Koszyce** [56]	POL		GAC	2880-2776 (as reported)	Male (1.5-2 yr); male (2-2.5 yr); male (5-6 yr); female (13-14 yr); female (15-16 yr); male (16-17 yr)	9 adults, incl. 5 females and 4 males	While described as a mass grave, this burial pit, containing the remains of 15 men, women and children, was richly furnished. The bodies were carefully laid out according to biological relatedness, with children buried next to their biological parents and siblings	Several 1st and 2nd degree relationships (incl. mother-son, mother-daughter, father-son, siblings).. Possible evidence for "extended family", traces of violence incl. lethal injuries [56]
36	**Künzing** [57]	DEU	Grave 2	CWC?		Indet. (3 yr).	Female (late adult)	Adult in flexed position cradling the child, 2 beakers, 2 stone axes, copper or bronze arm spiral	
37	**Kut** [58]	UKR	Kurgan 3, Grave 4	KAT		2 Indet.	Female	Adult in flexed position on the left side. One child to the left of the adult, in flexed position on the right side. The other child buried in another section of the pit, in flexed position on the left side, clay bead, several tubular bone beads	
38	**Kuz'mino** [59]	RUS	Grave 1	FAT		Neonate	Male (25 yr)	Adult in flexed position on the left side, facing the child, right hand on the child, axe head, perforated animal teeth and tubular bones, shell beads and fragments, faunal remains incl. pig hooves	
39	**La Almoloya** [60]	ESP	Grave 21	EBA	2000-1750 (as reported)	Female neonate	Female (30-35 yr)	Adult individual was deposited holding neonate to the right side of her chest	1st degree related, mother-daughter; shared mtDNA-haplogroup H1j [60]

Site ID	Site	Countr y code	Barrow, Grave No	Culture	Date (calBC, 2σ)	Children	Adults	Body position, grave goods	Note, incl. degree of relatedness (as reported)
39	**La Almoloya** [60]	ESP	Grave 85	EBA		Female neonate	Female	Adult individual was deposited holding neonate to her chest	[60]
40	**La Atalayuela** [61,62]	ESP		BBC				Collective burial; 20% of inhumed individuals subadult. No clear relation between subadult and adult individuals.	
41	**La Bastida** [60]	ESP	Grave 6	EBA		Male neonate	Male (25-30 yr)	Individuals buried in pithos	Individuals not genetically related, Child possible successive inhumation [60]
42	**La Salmedina** [48]	ESP	Grave 1	BBC		Indet	Male	Ciempozuelos bowl, Bell Beaker, bowl	
42	**La Salmedina** [48]	ESP	Grave 2-3, small chamber	BBC		Indet	Female	Ciempozuelos bowl, plain bowl, tanged dagger, awl, V-perforated bone button with the adult	
43	**Lauda-Königshofen** [63,64]	DEU	Grave 9	CWC		Neonate (0-3 months); male? (4 yr);	Female (30-35 yr)	Adult in flexed position on the left side, cradling the infant. Child in flexed position on the right side, facing the opposite direction 2 ceramic vessels, bone awl, faunal remains	
43	**Lauda-Königshofen** [63,64]	DEU	Grave 20	CWC		Foetus (8-9 months *in utero*)	Female (35-45 yr)	Adult in flexed position on the left side, skull displaced, foetus found in the pelvic area of the adult, ceramic vessel, flint blade, bone awl, canid teeth	Disturbed
43	**Lauda-Königshofen** [63,64]	DEU	Grave 29	CWC		Probably female (3-4 yr)	Female (25-30 yr)	Adult in flexed position on the left side, facing the child. The child is in flexed position on the left side, facing away from the adult, ceramic vessel, worked bone	

Site ID	Site	Country code	Barrow, Grave No	Culture	Date (calBC, 2σ)	Children	Adults	Body position, grave goods	Note, incl. degree of relatedness (as reported)
43	**Lauda-Königshofen** [63,64]	DEU	Grave 30	CWC		Indet. (9-12 months); Probably female (4-6 yr); indet. (13-16 yr)	Probably female (14-18 yr)	Adult probably in flexed position on the left side, facing the older child, who may be buried in the same position, thus facing away from the adult, decorated ceramic vessel, flint blade	Disturbed
43	**Lauda-Königshofen** [63,64]	DEU	Grave 63	CWC		Neonate (0-6 months); indet. (9-13 yrs)	Female (~40 yr)	Adult in flexed position on the left side. Position of the neonate and the child unclear, ceramic vessel, flint blade, bone awl, faunal remains	
43	**Lauda-Königshofen (“Reißwag”)** [65]	DEU	Feature 567	CWC		Indet. (1,5 yr); indet. (8 yr); indet. (15 yr)	-	Subadult in flexed position on the left side, holding the younger child. The older child is behind the subadult, in flexed position on the left side, no grave goods	
44	**Leau, Osternienburg, Wulfen** [24]	DEU	Feature 3001	UNE		Probably female (12-16 yr)	Probably female (18-20 yr); indet. (18-21 yr); probably male (24-28 yr)	Four individuals in a stone cist	Two pairs of possibly maternally related individuals [24]
45	**Łęki Małe** [66]	POL	Barrow 4, graves 1-2	BBC, UNE	c. 2300–2000 BC (as reported)	Female (13-16 yr)	Male (30-40 yr)	Faunal remains, ochre, ceramic sherds, flint	
46	**Léry, Les Petits Près 2, Eure** [67]	FRA	Grave 5	BBC	2568-2142	2 full-term foetuses	Female	Adult in contracted position, facing the two foetuses, bones of Salmonidae	

Site ID	Site	Countr y code	Barrow, Grave No	Culture	Date (calBC, 2σ)	Children	Adults	Body position, grave goods	Note, incl. degree of relatedness (as reported)
47	**Mains of Melgund, Angus** [68]	GBR	Stone cist grave	BBC	1886-1641	Indet. (7-9 yr)	Male (25-35)	Food Vessel, containing organic residue of cereal grains	
48	**Mamai-Hora** [69]	UKR	Grave 14	YAM?		Indet.	Female (40-45 yr)	Adult in supine position with flexed legs. Child to the right of the adult, in supine position with flexed legs, ceramic vessel, flintstone, shells	
49	**Mar'yanskii** [70]	UKR	Kurgan 1, Grave 7	YAM		Indet.	Male	Adult in supine position with flexed legs. Child to the right of the adult, in supine position with flexed legs, ochre	
50	**Marschwitz / Marszowice** [71]	POL		CWC		2 children	Female	Adult in flexed position on the right side. Both children face the adult and are buried in flexed position on the left side	
51	**Mortimer's Barrow 4, Painsthorpe Wold** [44,72]	GBR	Barrow 4	BBC		Indet. (8-12 yr)	Adult "aged"	2 beakers, 1 stood at each shoulder.	
52	**Nerushai** [73]	UKR	Kurgan 9, Grave 9	YAM		Indet.	Female	Adult in supine position with flexed legs, right arm extended over the child. The child is to the right of the adult, in flexed position on the left side, ceramic sherds, ochre	Two individuals buried with a dog
53	**Oechlitz** [74]	DEU	Feature 25645	BBC, CWC	c. 2470-2300 (as reported)	Indet. (3.5-6.5); indet. (4-8 yr); indet. (11-17 yr); probably male (13-19 yr)	Indet. (35-45 yr)	Three skeletons are NE-SW oriented, two are NW-SE oriented, suggesting a mixed BBC-CWC burial. The older adolescent and the adult are buried in flexed position on the right side, with the head to the SW. The latter is hugging a child, who is also buried in flexed position. Another subadult and a child are buried in flexed position at the feet of the adult, with the head to the SE. Polished stone axe, adze, Corded Ware vessel, shells, possibly a copper/bronze armband, bone beads, dog teeth, boar tooth	

Site ID	Site	Country code	Barrow, Grave No	Culture	Date (calBC, 2σ)	Children	Adults	Body position, grave goods	Note, incl. degree of relatedness (as reported)
54	**Okunev Ulus** [75]	RUS	Grave 1	OKU		Indet.	Female	Both individuals were buried in supine position with flexed legs and heads oriented toward NNW. 2 bronze earrings, copper braids, copper scales	
55	**Pervomaiskii** [76]	UKR	Group 1, Kurgan 1, Grave 9	YAM		Indet.	Indet.	Adult in supine position with flexed legs. Child to the left of the adult, in flexed position on the right side, ochre, wood	
55	**Pervomaiskii** [76]	UKR	Group 1, Kurgan 3, Grave 4	KAT		Indet.	2 Adults	One adult in supine position with flexed legs. The child is buried at the feet of the adults, position unclear, 2 ceramic vessels	
56	**Plötzkau** [24]	DEU	Feature 30	UNE		Male? (2 yr)	Probably male (19-23 yr)		
56	**Plötzkau** [24]	DEU	Feature 31	UNE		Indet (2-3 yr); indet (5 yr); male (15-17 yr)	Male (17-21 yr); female (30-35 yr)	Circular pit with the remains of five individuals	Female described as potential “mother” of the children [24]
57	**Pont-du-Château/Chazal, Auvergne** [77]	FRA		BBC		Indet.	Probably female	Adult and child buried face to face	
58	**Röcken** [24]	DEU	Feature 370	UNE		Indet. (12-14 yr); indet (12-14 yr)	Female; male	Four skeletons in tightly contracted position on top of each other in a narrow pit	The female and both children share the same mtDNA haplotype U8b1a1 [24]
59	**Sal’dyar-1** [78]	RUS	Kurgan 42	AFA		Indet. (6-7 yr)	Female (35-40 yr)	Adult female in supine position with flexed legs. Child to the right of the adult, in supine position with flexed legs, ceramic vessel	

Site ID	Site	Country code	Barrow, Grave No	Culture	Date (calBC, 2σ)	Children	Adults	Body position, grave goods	Note, incl. degree of relatedness (as reported)
60	**Salhir** [79]	UKR	Kurgan 1, Grave 2	YAM		Indet.	Female	Adult in supine position with flexed legs, facing the child, who is buried to the left in flexed position, charcoal	
61	**Schleinbach** [39,80]	AUT	Grave 60	UNE	1919-1701	Indet. (3–4 yr); indet. (8–9 yr); indet. (12–14 yr)	Male (27–35 yr)	Adult in an extended position, with open legs and folded arms, placed directly on top of two children. Children buried left and right of the adult, with their head facing away from the adult. Reuse of an old storage pit, a few ceramic sherds and faunal remains, 2 complete EBA jugs higher in the fill	Signs of violence, two of the children share the same mtDNA haplogroup U2e3a [80]
62	**Selgas** [81]	LAT		CWC		Indet. (1-1,5 yr)	Female (40-45 yr)	Adult in supine position, legs flexed on the right, position of arms indeterminable due to disturbance; Subadult buried at the adult's feet, heavily disturbed, position indet.; flint knife, glycymeris shell, bone chisel, partial antler, unworked bones, bone awls, large amphora, other small ceramic sherds	
63	**Serbitz** [24]	DEU	Feature 142	UNE		Indet. (7-8 yr); indet. (7-9 yr)	Male (35-40 yr); male (40-45 yr)	Faunal remains, ceramic sherds, three salt panes	
64	**Smeeni** [82]	ROU	Grave 19	YAM		Indet.	Male (30-60 yr)	Adult in supine position with flexed legs. Subadult to the left of the adult, in supine position with flexed legs, mat impressions, ochre, shell-decorated ceramic, necklace of perforated pig canines, animal	
65	**Spreitenbach -Moosweg** [83,84]	CHE		CWC	c. 2830-2350 (as reported)	Foetus; neonate; indet. (12-15 yr); male (16-19 yr)	9 adults, incl. 4 women and 5 men	Twelve individuals in a burial chamber, all primary inhumations. Adults in flexed position on one side. Neonate near the arm of adult female (50-70 yr). Older adolescent lies directly under younger one, in flexed position on the right side. Younger adolescent probably on the left side. 13 artefacts, no ceramic	Timber structure? Shared mtDNA lineages between some adults [85]

Site ID	Site	Country code	Barrow, Grave No	Culture	Date (calBC, 2σ)	Children	Adults	Body position, grave goods	Note, incl. degree of relatedness (as reported)
66	**Stetten a. D. Donau** [86,87]	DEU	Grave 3	CWC	2882-2585	Neonate (9-10 months)	Female (30 yr)	Silex blade, 2 bone points, fragment of a sharpening stone, faunal remains	
67	**Święte** [30]	POL	Grave 43	CWC	2571-2305	Indet. (7-9 yr)	Male (40-45 yr); female (44-55 yr)	Adult male in flexed position on the right side. Scattered and incomplete remains of a child and an adult woman in a niche construction, amphora, beaker, 3 cups, 4 axes, fire-flint, 2 flakes, whetstone	1st degree relative (father of the 6-7 yr male child buried in grave 40A) [30]
68	**Szarbia Zwierzyniecka** [88]	POL	Grave 23/IX	BBC		Indet. (2-4 yr)	Male (60+ yr)	Clay pot, faience bead, fragments of boar-tusk pendant	
69	**Tauberbischofsheim -Dittigheim** [14,63,89]	DEU	Grave 17	CWC	2580-2468	Indet. (15-17 yr)	Female (30-40 yr)	Adult in flexed position on the right side. Behind her in the grave, remains of a subadult in flexed position on the right side, decorated s-shaped beaker, two flint tools, bone tool, pierced shell disc	
69	**Tauberbischofsheim -Dittigheim** [63]	DEU	Grave 18	CWC		Indet.; indet.	Female (30 yr)	Adult and children buried side-by-side	
69	**Tauberbischofsheim -Dittigheim** [14,63,89]	DEU	Grave 21	CWC	2474-2236	Indet. (3 yr); indet. (7-8 yr); Indet. (13-16 yr)	-	The subadult is in flexed position on the right side, facing the older child, who is in flexed position on the left side. The younger child, though fragmented, was probably buried in flexed position on the right side. A beaker was deposited at the feet of the subadult.	
69	**Tauberbischofsheim -Dittigheim** [14,63,89]	DEU	Grave 25	CWC	2558-2342	Indet. (1-1.5 yr); indet. (8-9 yr); male (16-18 yr)	Female (25-30 yr); female (30-40 yr); male (30-40 yr)	Six individuals in a square pit with rounded corners, deposited in at least two phases. All primary inhumations on one side, except adult male, whose bones have been moved to make space for other inhumations, pierced animal teeth	

Site ID	Site	Countr y code	Barrow, Grave No	Culture	Date (calBC, 2σ)	Children	Adults	Body position, grave goods	Note, incl. degree of relatedness (as reported)
70	**Tauberbischofsheim -Impfingen** [14,63,89]	DEU	Grave 6	CWC	2121-1624	Neonate (4-6 months); indet. (14-16 yr)	Female (20-30 yr); male (20-30 yr)	Four individuals in a rectangular burial pit. Man in flexed position on the left side, partly overlapping juvenile in flexed position on the left side, both facing the same direction. The woman is in flexed position on the right side, facing the neonate, who is buried on the left side in her arms, undecorated vessel	
70	**Tauberbischofsheim -Impfingen** [14,63,89]	DEU	Grave 10	CWC	2342-1885	Male (10-14 yr)	Male; male (old)	Two adult male and one adolescent in a grave pit, disturbed. The adults are in flexed position on the left side, facing the same direction. The adolescent is in-between the two adults, on the right side. Beaker, footed bowl, miniature vessel, flint blade	
71	**Tas Khazaa** [90]	RUS	Barrow 4	OKU		Indet. (infant)	Male; 2 females	Infant buried beside central female individual. Female individual embraces infant, this is possibly due to a shift of arms of the female individual caused by grave collapse. All individuals deposited in flexed supine position, heads toward SW. Copper axehead, marmor spheres, incense bowl, copper needle case, animal teeth, grindstone, Afanasievo-type vessel, stone pestle	
71	**Tas Khazaa** [90]	RUS	Barrow 5	OKU		Indet. (infant)	Male (old); female	All individuals buried in flexed supine position. Infant placed between male and female individual. 12 flintstone arrowheads, 13 perforated sable jawbones, marmot teeth, bronze ring	
72	**Tiefbrunn** [91,92]	DEU		CWC	c. 2880-2498 (as reported)	Female (infant)	Male (old); Male	Two adult males and one infant female. Older male has non-local Sr-ratios, male adult and female infant show local Sr-ratios. All three individuals show signs of trauma on the skull. Older male was buried with steppe-type hammer headed pin	All three individuals not related on maternal side [92]
73	**Trebnitz** [71]	DEU	Grave 6	CWC		Indet.	Female		

Site ID	Site	Country code	Barrow, Grave No	Culture	Date (calBC, 2σ)	Children	Adults	Body position, grave goods	Note, incl. degree of relatedness (as reported)
74	**Trumpington, Cambridgeshire** [93]	GBR	F.1596	BBC	2193-1982	Female (16-18 yr)	Male (17-20 yr)	Decorated beaker at head of each individual; clay disk	
75	**Unterhautzenthal** [39,94]	AUT	Grave 38	UNE		Neonate; female (14-15 yr)	-	Subadult in supine position, ceramic sherds, faunal remains	
75	**Unterhautzenthal** [39,94]	AUT	Grave 95	UNE		Indet. (5 yr); indet. (3-4 yr)	Female (35-45 yr)	Female facing the younger child. Another child behind the female's legs, with an arm ring, a bone ring	
75	**Unterhautzenthal** [39,94]	AUT	Grave 103	UNE		Indet. (3 yr)	Female (16-20 yr)		
75	**Unterhautzenthal** [39,94]	AUT	Grave 109	UNE		Neonate	Female (50-60 yr)		
75	**Unterhautzenthal** [39,94]	AUT	Grave 116	UNE		Indet. (4-6 yr)	Female (45-60 yr)		
75	**Unterhautzenthal** [39,94]	AUT	Grave 122	UNE		Neonate	Female (17-20 yr); male (35-40 yr)		
76	**Unterwölbling** [39,95]	AUT	Grave 14	UNE		Indet (1-2 yr); indet (3-6 yr);	Male (60 yr)		

Site ID	Site	Country code	Barrow, Grave No	Culture	Date (calBC, 2σ)	Children	Adults	Body position, grave goods	Note, incl. degree of relatedness (as reported)
76	**Unterwölbling** [39,95]	AUT	Grave 26	UNE		Indet (5 yr); indet (14 yr)	-		
77	**Valle de las Higueras** [48,96]	ESP	Cave 3, central niche	BBC		Indet (2-4 yr); indet (7 yr)	Female	Plain bowl, carinated bowl and Bell Beaker	
78	**Västerbjers** [97,98]	SWE	Grave 67:1	PWC	2877-2496	Indet.	Female	Child laid on left arm of woman, both in supine position. Superimposed on grave 67:2, partially disarticulated bones from an older child and an adult man, dog tooth pendants (teeth from at least 32 dogs)	
78	**Västebjers** [97,98]	SWE	Grave 67:2	PWC		Indet.	Male		
78	**Västerbjers** [97,98]	SWE	Grave 82	PWC		Indet. (young)	Male (young)	Faunal remains	
79	**Verkhnii Askiz I** [99]	RUS	Kurgan 2, Grave 35	OKU		Indet. (7-8 yr)	Male? (35-45 yr)	Both individuals buried in supine position with flexed legs, oriented toward W. Adult's left arm stretched out and placed on chest of subadult. Both individuals were buried without head and neck.	
80	**Vikletice** [31,100]	CZE	Grave 2/1964	CWC		Male? (juvenile)	Female?	Adult in flexed position on the left side, juvenile on the right side	
80	**Vikletice** [31,100]	CZE	Grave 21/1965	CWC		Indet. (infans II)	Female?	Adult and child buried side by side	

Site ID	Site	Country code	Barrow, Grave No	Culture	Date (calBC, 2σ)	Children	Adults	Body position, grave goods	Note, incl. degree of relatedness (as reported)
80	**Vikletice** [31,100]	CZE	Grave 47/1964	CWC		Indet. (4-6 yr)	Female? (30-40 yr)	Adult in flexed position on the left side, child on the right side	
80	**Vikletice** [31,100]	CZE	Grave 110/1963	CWC		Indet. (4-5 yr)	Male? (20-30 yr)	Adult in flexed position on the right side, child on the left side	
80	**Vikletice** [31,100]	CZE	Grave 143/1963	CWC		Indet. (4-5 yr)	Male? (30-40 yr)	Position of both individuals unclear	
80	**Vikletice** [31,100]	CZE	Grave 155/1963	CWC		Female? (14-15 yr); male? (16 yr)	-	Female (?) in flexed position on the left side, male (?) on the right side	
81	**Wangenheim (Gotha)** [71]	DEU	Grave 7	CWC		Indet.	Female	Both in extended position	
82	**Węgrzce, Comm. Zielonki** [101]	POL	Grave 3/2016	CWC	2561-2299	Indet. (4-5 yr)	Male (38-47 yr)	Beaker, stone shaft axe, bone tool, silex knife insert, 3 silex flakes	
83	**Weimar** [71]	DEU		CWC		Indet.	adult	Adult and child buried with head to opposite direction	
84	**Willerby, Yorkshire** [41]	GBR	Barrow 3	BBC?		Indet. (4 yr)	Female (60+ yr)	No grave goods. Part of larger burial	
85	**Wojciechowice** [40]	POL	Grave 24	CWC		Indet. (infans II)	Female? (p. juv.)	Niche grave	

Site ID	Site	Country code	Barrow, Grave No	Culture	Date (calBC, 2σ)	Children	Adults	Body position, grave goods	Note, incl. degree of relatedness (as reported)
86	**Yazykovo I** [102]	UKR	Grave 20	VOL		Indet.	Female	Adult in supine position. Child to the left of the adult, in supine position, ceramic, charcoal, faunal remains incl. bear phalanges, bear molar	
87	**Żerniki Górne** [40]	POL	Grave 33	CWC		Indet. (juvenile)	Male (maturus)	Niche grave	
87	**Żerniki Górne** [40]	POL	Grave 67	CWC		Indet. (infans II)	Male (maturus)	Niche grave	
87	**Żerniki Górne** [40]	POL	Grave 78	CWC		Female (juvenile)	Female	Niche grave. Adult in flexed position on the right side, juvenile on the left side	
87	**Żerniki Górne** [40]	POL	Grave 90	CWC		Indet. (infans I)	Female (juv./ad.)	Niche grave	
87	**Żerniki Górne** [40]	POL	Grave 97	CWC		Indet. (infans I)	Female (maturus)	Niche grave	
87	**Żerniki Górne** [40]	POL	Grave 120	CWC		Indet. (infans I)	Female (maturus)	Niche grave	
87	**Żerniki Górne** [40]	POL	Grave 130	CWC		Indet. (infans II)	Male (juv./ad.)	Niche grave. Adult in flexed position on the left side, child on the right side	
87	**Żerniki Górne** [40]	POL	Grave 139	CWC		Indet. (infans II)	Female	Niche grave	

Site ID	Site	Country code	Barrow, Grave No	Culture	Date (calBC, 2σ)	Children	Adults	Body position, grave goods	Note, incl. degree of relatedness (as reported)
88	**Złota, st. 59** [40]	POL		CWC		Indet. (infans I); indet. (infans II); indet. (infans II)	Female (ad./mat.)		

Supplementary Information 3. Map of Eurasia
(IBD sharing patterns)

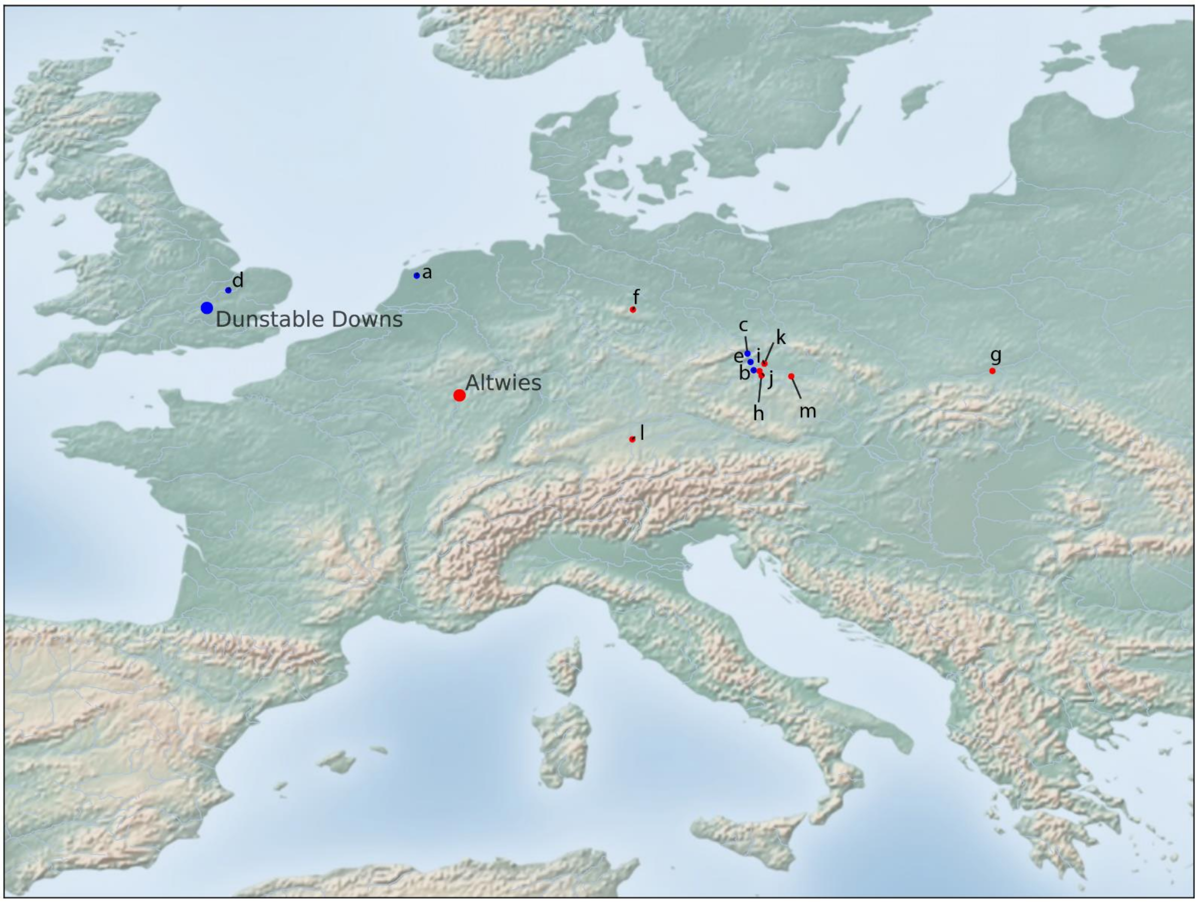

Figure S2. Location of archaeological sites in which individuals were identified sharing at least one IBD segment of ≥16cM with individuals buried at Altwies (red dots) and Dunstable Downs (blue dots). For further information about the sites and results, see SI Table 'hapROH'. Matplotlib Basemap Toolkit 1.3.8 and Python 3.11 were used to produce the map. As map background, a display shaded relief image (from http://www.shadedrelief.com) was used. Legend: a. De Tuithoorn (2192-1887 cal. BC); b. Brandýsek (2462-2298 cal. BC); c. Trmice (2862-2573 cal. BC); d. Needingworth Quarry, Over Narrows (2199-1982 cal. BC); e. Radosevice (2403-2199 cal. BC); f. Benzingerode (2343-2137 cal. BC); g. Koszyce, site 3 (2891-2674 cal. BC); h. Prague 5, Jinonice, Butovická Street (2287-2140 cal. BC); i. Vliněves (~2300-2100 BC); j. Velké Přílepy (~2500-1900 BC); k. Vliněves (2572-2351 cal. BC); l. Augsburg (~2500-2000 BC); m. Kolín II (2463-2296 cal. BC).

Supplementary Information 4. Anthropology of Dunstable Downs

The skeletal remains of the two individuals from Dunstable Downs Barrow 8 (Tumulus 6 according to Worthington Smith's labelling) were examined at Luton Culture Trust. Based on morphological analysis of the cranium, the adult individual (Fig. S3) is a woman. Age at death was estimated at 18-25 years of age, by observing the ectocranial suture obliteration and dental wear. The health of the woman was good, with no visible signs of metabolic deficiencies. There are no signs of trauma or pathological conditions. The right mandibular branch, the only one to be preserved, has three molars, two premolars and the right canine still in situ. The crown of the third molar is completely erupted but has not quite reached the same occlusal surface as the second molar. No dental tartar is present, but a small non-penetrating occlusal caries is present on the third molar. Pelvic bones were present in their entirety, glued together in a fixed anatomical position. However, research has revealed that these bones do not belong to the excavated skeleton but were added when the remains were prepared for display in the 1920s, as the original pelvis was in a 'shattered and soft condition' ([103], 37). They should therefore be discounted from analysis.

The second individual from the Dunstable burial is a subadult (Fig. S4). Age at death was estimated at between 4 and 8 years of age, based on dental eruption and bone epiphysis fusion. The skeletal remains are very fragmented. This is not surprising, as subadult skeletal remains are small, thin and often not completely mineralized, rendering them difficult to recover from the archaeological record. Again, there are no visible signs of skeletal trauma or pathologies. Only three teeth remain, the two mandibular deciduous molars on the right side and the mandibular first permanent molar in eruption on the same side. All three teeth are in situ. The rest of the mandibular teeth are lost post-mortem. There are no signs of dental pathologies.

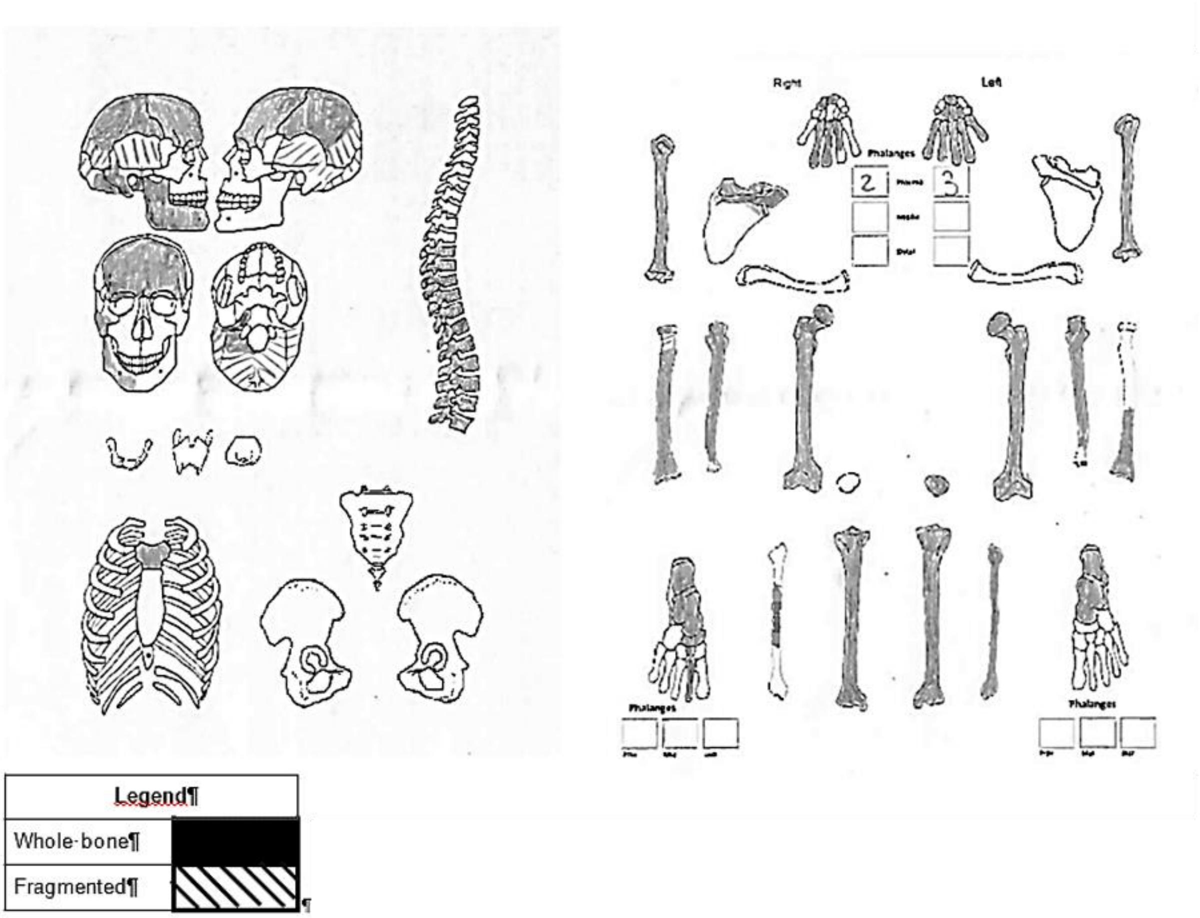

Figure S3: Adult woman skeleton from Dunstable Downs Barrow 8. Picture by Nicoletta Zedda, using the recording sheets from BABAO Updated Guidelines for Standards for Recording Human Skeletal Remains.

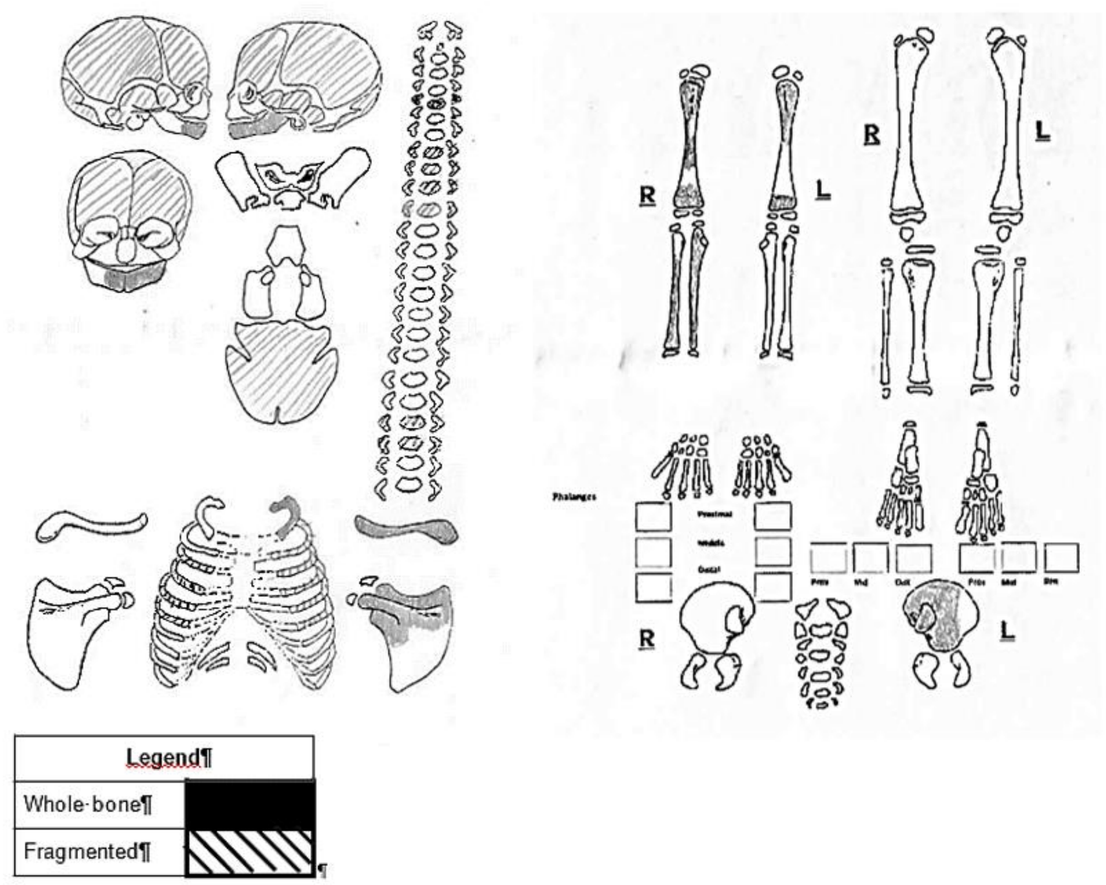

Figure S4: Child skeleton from Dunstable Downs Barrow 8. Picture by Nicoletta Zedda, using the recording sheets from BABAO Updated Guidelines for Standards for Recording Human Skeletal Remains.

Supplementary References

1. Reimer PJ, Austin WEN, Bard E, Bayliss A, Blackwell PG, Ramsey CB, et al. The IntCal20 Northern Hemisphere Radiocarbon Age Calibration Curve (0–55 cal kBP). Radiocarbon. 2020;62: 725–757.

2. Bronk Ramsey C. OxCal v4. 4.4. Available at: Retrieved from https://c14 arch ox ac uk. 2021.

3. Toussaint M, Le Brun-Ricalens F, Hauzeur A. Les deux sépultures campaniformes d'Altwies, Op dem Boesch (Grand-Duché de Luxembourg) : méthodologie, données anthropologiques préliminaires et essai de caractérisation des pratiques sépulcrales. Bulletin de la Société préhistorique luxembourgeoise. 2001;23-24: 249–284.

4. Le Brun-Ricalens F, Hauzeur A, Toussaint M, Jost C, Valotteau F. Les deux sépultures campaniformes d'Altwies, Op dem Boesch (Grand-Duché de Luxembourg): matériel archéologique et contexte régional. Bull Soc Préhist Luxembourgeoise. 2001;23: 285–300.

5. Smith WG. Man, the Primeval Savage: His Haunts and Relics from the Hill-tops of Bedfordshire to Blackwall. E. Stanford; 1894.

6. Dyer J. The Five Knolls and Associated Barrows at Dunstable, Bedfordshire. Beds Arch. 1991.

7. Ulrich H. Ein Zonenbechergrab von Achenheim im Elsass. Germania: Anzeiger der Römisch-Germanischen Kommission des Deutschen Archäologischen Instituts. 1942;26: 175–177.

8. Грязнов МП. Афанасьевская культура на Енисее. С.-Петербург: Дмитрий Буланин; 1999.

9. Вадецкая ЭБ, Поляков АВ, Степанова НФ. Свод памятников афанасьевской культуры. Барнаул: Азбука; 2014.

10. Buck H. Das Hockergrab von Althausen, Kreis Mergentheim. Tübingen; 1946.

11. Jungsteinzeit im Taubertal. Das Hockergrab von Althausen. In: Schloss Mergentheim, Baden-Württemberg [Internet]. [cited 3 Jun 2023]. Available: https://www.schloss-mergentheim.de/erlebnis-schloss-garten/verborgene-schaetze/hockergrab-von-althausen

12. Mittnik A, Massy K, Knipper C, Wittenborn F, Friedrich R, Pfrengle S, et al. Kinship-based social inequality in Bronze Age Europe. Science. 2019;366: 731–734.

13. Engelhardt B. Bemerkungen zur Schnurkeramik in Straubing und im Landkreis Straubing-Bogen. Jahresbericht des Historischen Vereins für Straubing und Umgebung. 1998;100: 27–84.

14. Furholt M. Die absolutchronologische Datierung der Schnurkeramik in Mitteleuropa und Südskandinavien. Bonn: Habelt, R; 2003.

15. Kapps K, Bailloud G. Découverte fortuite d'une sépulture chalcolithique à la ferme de Champagne: Commune d'Augy (Yonne). Bulletin de la Société préhistorique de France. 1960;57: 476–479.

16. Salanova L. La question du Campaniforme en France et dans les îles anglo-normandes : Productions, chronologie et rôles d'un standard céramique. PARIS: CTHS: Société Préhistorique Française; 2000.

17. Feustel R, Bach H, Gall W, Teichert M. Beiträge zur Kultur und Anthropologie der mitteldeutschen Schnurkeramiker. Alt-Thuringen. 1966;8: 20–170.

18. Попандопуло ЗХ. Курганы эпохи меди-бронзы Бабурского могильника. ДСПК–Запорожье: Изд-во ЗГУ. 1993;4: 79–101.

19. Бадер ОН. Балановский могильник. Москва: Изд-во АН СССР; 1963.

20. Кубарев ВД, Черемисин ДВ, Слюсаренко ИЮ. Бике-I, II: погребальные памятники афанасьевской культуры на Средней Катуни. Древности Алтая: Известия лаборатории археологии. 2001. Available: https://e-lib.gasu.ru/da/archive/2001/N6/st3.pdf

21. Hnízdová I, Šimůnek J. Hrob se šňůrovou keramikou v Blšanech. Archeologické rozhledy. 1955;7: 577–582.

22. Turek J. Being a Beaker child. The position of children in Late Eneolithic society. In Memoriam Jan Rulf. 2000; 424–438.

23. Крайнов ДА. Болшневский могильник фатьяновской культуры. Памятники древнейшей истории Евразии. 1975; 173–180.

24. Knipper C, Fragata M, Nicklisch N, Siebert A, Szécsényi-Nagy A, Hubensack V, et al. A distinct section of the Early Bronze Age society? Stable isotope investigations of burials in settlement pits and multiple inhumations of the Únětice culture in central Germany. Am J Phys Anthropol. 2016;159: 496–516.

25. Clarke DL. Beaker Pottery of Great Britain and Ireland. University Press; 1970.

26. Liesau von Lettow-Vorbeck C, Blasco C, Ríos P, Vega J, Menduiña R, Francisco BLANCO J, et al. A space for the living and the dead: The Chalcolithic ditched enclosured settlement of Camino de las Yeseras (San Fernando de Henares, Madrid). Ediciones Complutense; 2008 [cited 24 Mar 2023]. Available: https://revistas.ucm.es/index.php/CMPL/article/download/CMPL0808110097A/29274/0

27. Olalde I, Brace S, Allentoft ME, Armit I, Kristiansen K, Booth T, et al. The Beaker phenomenon and the genomic transformation of northwest Europe. Nature. 2018;555: 543.

28. Maurandi JL, Martínez ML, Martínez FR, Fernández AA. El enterramiento múltiple, calcolítico, de Camino del Molino (Caravaca, Murcia). Metodología y primeros resultados de un yacimiento excepcional. Trabprehist. 2009;66: 143–159.

29. Maurandi JL, Martínez MVL, Martínez FR. Un excepcional sepulcro del Calcolítico: Camino del Molino (Caravaca de la Cruz). XX Jornadas de Patrimonio Cultural de la Región de Murcia: Cartagena, Cieza, Águilas, Puerto Lumbreras y Murcia, 6 de octubre al 3 de noviembre 2009. Tres Fronteras; 2009. pp. 205–219.

30. Linderholm A, Kılınç GM, Szczepanek A, Włodarczak P, Jarosz P, Belka Z, et al. Corded Ware cultural complexity uncovered using genomic and isotopic analysis from south-eastern Poland. Sci Rep. 2020;10: 6885.

31. Turek J. The social meaning of multiple burials in the Corded Ware culture. Přehled výzkumů. 2023;64: 77–83.

32. Alexandrov S. Site 3 near Chudomir, Loznitsa Municipality. In: Popov H, Chukalev K, Dimitrova Y, Kecheva N, Damyanov M, Ivanova N, et al., editors. Stream through time Rescue archaeological investigations along the expansion of the gas transmission infrastructure of Bulgartransgaz EAD. Sofia: National Archaeological Museum Catalogues; 2020. pp. 462–471.

33. Максименков ГА. Могильник Черновая VIII-эталонный памятник окуневской культуры. Памятники окуневской культуры. 1980. pp. 3–26.

34. Haak W, Brandt G, de Jong HN, Meyer C, Ganslmeier R, Heyd V, et al. Ancient DNA, Strontium isotopes, and osteological analyses shed light on social and kinship organization of the Later Stone Age. Proc Natl Acad Sci U S A. 2008;105: 18226–18231.

35. Meyer C, Brandt G, Haak W, Ganslmeier RA, Meller H, Alt KW. The Eulau eulogy: Bioarchaeological interpretation of lethal violence in Corded Ware multiple burials from Saxony-

Anhalt, Germany. Journal of Anthropological Archaeology. 2009;28: 412–423.

36. Meyer C, Ganslmeier R, Dresely V, Alt KW. New approaches to the reconstruction of kinship and social structure based on bioarchaeological analysis of Neolithic multiple and collective graves. In: Kolář J, Trampota F, editors. Theoretical and Methodological Considerations in Central European Neolithic Archaeology. Oxford: Bar International Series; 2012. pp. 11–23.

37. Berner M, Wiltschke-Schrotta K. Das frühbrozezeitliche Gräberfeld von Franzhausen I, Niederösterreich 1. Allgemeine Übersicht. Anthropol Anz. 1992;50: 1–12.

38. Neugebauer-Maresch C, Neugebauer J-W. Franzhausen: das frühbronzezeitliche Gräberfeld I. Horn: Ferdinand Berger & Söhne; 1997.

39. Rebay-Salisbury K. 3. Personal Relationships between Co-buried Individuals in the Central European Early Bronze Age. AmS-Skrifter. 2018. Available: https://journals.uis.no/index.php/AmS-Skrifter/article/view/208

40. Włodarczak P. Kultura ceramiki sznurowej na Wyżynie Małopolskiej. Kraków: Instytut Archeologii i Etnologii Polskiej Akademii Nauk; 2006.

41. Greenwell W. British Barrows: A Record of the Examination of Sepulchral Mounds in Various Parts of England. Clarendon Press; 1877.

42. Тощев ГН, Шахров ГИ. Раскопки курганной группы у с. Григорьевка Запорожской области. Древности степного Причерноморья и Крыма. 1992;3: 49–70.

43. Häusler A. Vergleichende Untersuchungen zu den Bestattungssitten Mittel- und Osteuropas seit der frühen Bronzezeit. journals.ub.uni-heidelberg.de; 2009 [cited 19 Jan 2023]. Available: https://journals.ub.uni-heidelberg.de/index.php/jsmv/article/download/72126/65524

44. Mortimer JR. Forty years' researches in British and Saxon burial mounds of East Yorkshire: including Romano-British discoveries, and a description of the ancient entrencements of a section of the Yorkshire wolds. A. Brown and sons, limited; 1905.

45. Lefebvre A, Franck J, Veber C, Duval H. Les sépultures individuelles campaniformes en Lorraine: l'exemple de Pouilly (Moselle) et d'Hatrize (Meurthe-et-Moselle). Les sépultures individuelles campaniformes. 2011.

46. Jewitt LFW. Grave-mounds and Their Contents: A Manual of Archaeology, as Exemplified in the Burials of the Celtic, the Romano-British, and the Anglo-Saxon Periods. Groombridge and Sons; 1870.

47. Howarth E. Catalogue of the Bateman collection of antiquities in the Sheffield Public Museum. Dulau; 1899.

48. Herrero-Corral AM, Garrido-Pena R. The inheritors: Bell Beaker children's tombs in Iberia and their social context (2500–2000 cal BC). Journal of. 2019. Available: https://journal.equinoxpub.com/JMA/article/view/11886

49. Sjögren K-G, Olalde I, Carver S, Allentoft ME, Knowles T, Kroonen G, et al. Kinship and social organization in Copper Age Europe. A cross-disciplinary analysis of archaeology, DNA, isotopes, and anthropology from two Bell Beaker cemeteries. PLoS One. 2020;15: e0241278.

50. Kovalev AA, Erdenebaatar D. Discovery of new cultures of the Bronze Age in Mongolia according to the data obtained by the International Central Asian Archaeological Expedition. Current archaeological research in Mongolia. 2009; 149–170.

51. Ковалев АА, Эрдэнэбаатар Д. Афанасьевско-чемурчекская курганная группа Кургак гови

(Хуурай говь) и вопросы внешних связей афанасьевской культуры. Афанасьевский сборник. 2010; 91–108.

52. Honeychurch W, Rogers L, Amartuvshin C, Diimaajav E, Erdene-Ochir N-O, Hall ME, et al. The earliest herders of East Asia: Examining Afanasievo entry to Central Mongolia. Archaeological Research in Asia. 2021;26: 100264.

53. Wang C-C, Yeh H-Y, Popov AN, Zhang H-Q, Matsumura H, Sirak K, et al. Genomic insights into the formation of human populations in East Asia. Nature. 2021;591: 413–419.

54. Massy K. Die Gräber der Frühbronzezeit im südlichen Bayern. Untersuchungen zu den Bestattungs- und Beigabensitten sowie gräberfeldimmanenten Strukturen. Kallmünz /Opf.: Lassleben; 2018.

55. Stockhammer PW, Massy K. Mobility at the onset of the Bronze Age: A bioarchaeological perspective. Rethinking migrations in late prehistoric Eurasia. London, England: Oxford University Press; 2023. pp. 170–188.

56. Schroeder H, Margaryan A, Szmyt M, Theulot B, Włodarczak P, Rasmussen S, et al. Unraveling ancestry, kinship, and violence in a Late Neolithic mass grave. Proc Natl Acad Sci U S A. 2019;116: 10705–10710.

57. Engelhardt B. Die schnurkeramische Doppelbestattung von Künzing, Landkreis Deggendorf, Niederbayern. Arch Jahr Bayern. 1990;1989 (1990): 55–57.

58. Березовець ДТ. Розкопки курганного могильника епохи бронзи та скіфського часу в с. Кут. АП урср. 1960;9: 39–87.

59. Бадер ОН. Кузьминский могильник фатьяновского типа под Москвой. Археологический сборник Государственного Эрмитажа. 1962;5: 5–30.

60. Villalba-Mouco V, Oliart C, Rihuete-Herrada C, Rohrlach AB, Fregeiro MI, Childebayeva A, et al. Kinship practices in the early state El Argar society from Bronze Age Iberia. Sci Rep. 2022;12: 22415.

61. Maestu IB. La Atalayuela: fosa de inhumación colectiva del Eneolítico en el Ebro Medio. Príncipe de Viana. 1978;39: 381–422.

62. Maestu IB, Rupérez MTA. La tumba calcolítica de La Atalayuela, treinta y cinco años después. Saldvie: Estudios de prehistoria y arqueología. 2004; 85ß124.

63. Dresely V. Schnurkeramik und Schnurkeramiker im Taubertal. Stuttgart: Kommissionsverlag K. Theiss; 2004.

64. Menninger M. Die schnurkeramischen Bestattungen von Lauda-Königshofen. Steinzeitliche Hirtennomaden im Taubertal? Universität Tübingen. 2008. Available: https://ub01.uni-tuebingen.de/xmlui/handle/10900/49177

65. Keller R, Krausse DL. Von der Steinzeit zu den frühen Germanen: Landschafts-und Siedlungsentwicklung im Reißwag bei Königshofen, Stadt Lauda-Königshofen, Main-Tauber …. Archäologische Ausgrabungen in Baden. 2007; 94–97.

66. Kowiańska-Piaszykowa M. Cmentarzysko kurhanowe z wczesnej epoki brązu w Łękach Małych w Wielkopolsce. Poznań: Muzeum Archeologiczne w Poznaniu; 2008.

67. Mantel E. Les sépultures des Petits Prés et du Chemin des Vignes à Léry (Eure): Étude archéologique. Gall préhistoire. 1991;33: 185–192.

68. Rideout JS, Russell-White CJ. Mains of Melgund (NO 536 563). Tayside & Fife Archaeological

Journal. 4: 49–54.

69. Тощев ГН. Грунтовый могильник катакомбного времени на Мамай-горе. Древности Степного Причерноморья и Крыма. 1995; 32–40.

70. Березовець ДТ, Покровська СФ, Фурманська АІ. Кургани епохи бронзи поблизу с. Мар'янського. АП УРСР. 1960;9: 102–126.

71. Wilke G. Mutter und Kind. Ein Beitrag zur Frage des Mutterrechts. Mannus Zeitschrift für Vorgeschichte, Bd. 1929.

72. McLaren DP. Funerary rites afforded to children in earlier Bronze Age Britain: case studies from Scotland, Yorkshire and Wessex. PhD, The University of Edinburgh. 2011. Available: https://era.ed.ac.uk/handle/1842/35266

73. Шмаглий НМ, Черняков ИТ. Исследования курганов в степной части междуречья Дуная и Днестра (1964-1966 гг.). Материалы по археологии Северного Причерноморья. 1970;6: 5–115.

74. Fröhlich M, Becker M. Die endneolithische Mehrfachbestattung von Oechlitz, Saalekreis – Eine gemeinsame Grablege der Schnurkeramik- und Glockenbecherkultur. In: Meller H, Becker M, editors. Neue Gleise auf alten Wegen II Jüdendorf bis Gröbers Band II. Halle: Landesamt für Denkmalpflege und Archäologie Sachsen-Anhalt – Landesmuseum für Vorgeschichte; 2017. pp. 308–314.

75. Комарова МН. Погребения Окунева улуса. СА. 1947; 47.

76. Попандопуло ЗХ. Первомайский курганный могильник у с. Приморское. ДСПК-ТЛИ. 1992; 82–98.

77. Tchérémissinoff Y. Les sépultures simples et plurielles du Campaniforme et du Bronze ancien dans le Bassin rhodanien et ses zones d'influences. Oxford: British Archaeological Reports; 2006. p. 155p.

78. Ларин ОВ. Афанасьевская культура Горного Алтая: могильник Сальдяр-1. Барнаул: Алт. ун-та.; 2005.

79. Колосов ЮГ. Розкопки кургана № 1 на лівому березі Салгиру. Археологія Київ. 1961;12: 119–128.

80. Pany-Kucera D, Spannagl-Steiner M. Social Relations, Deprivation and Violence at Schleinbach, Lower Austria. Insights from an Interdisciplinary Analysis of the Early Bronze Age Human Remains. Archaeologia. 2020. Available: https://www.jstor.org/stable/27045105

81. Grasis N. The skaistkalnes Selgas double burial and the corded ware/rzucewo culture: A model of the culture and the development of burial practices. Lietuvos archeologija. 2007;31: 39–70.

82. Frînculeasa A, Simalcsik A, Preda B, Garvăn D. Smeeni-Movila Mare: monografia unui sit arheologic regăsit. Târgoviște: Editura Cetatea de Scaun; 2017.

83. Bleuer E, Huber H, Langgeger E. Das endneolithische Kollektivgrab von Spreitenbach im Kanton Aargau. Archéologie Suisse. 1999. Available: https://scholar.archive.org/work/uaimt73wwfdanmowbsjijrljv4/access/wayback/https://www.e-periodica.ch/cntmng?pid=ars-001:1999:22::261

84. Doppler T, Meyer C, Knipper C, Desideri J, Huber H, Hüster Plogmann H, et al. Gesamtheitliche Betrachtungen zum endneolithischen Kollektivgrab von Spreitenbach-Moosweg – eine integrative Synthese = Considérations globales à propos de la sépulture collective de Spreitenbach-Moosweg – une synthèse intégrativ. Spreitenbach-Moosweg (Aargau, Schweiz) : ein Kollektivgrab um 2500 vChr. Basel: Archäologie Schweiz; 2012. pp. 287–314.

85. Warnberg O, Alt KW. Molekulargenetische Analysen an den Bestattungen aus dem endneolithischen Kollektivgrab von Spreitenbach. In: Doppler T, editor. Spreitenbach-Moosweg (Aargau, Schweiz): ein Kollektivgrab um 2500 vChr. Basel: Archäologie Schweiz; 2012. pp. 158–169.

86. Wahl J, Dehn R, Kokabi M. Eine Doppelbestattung der Schnurkeramik aus Stetten an der Donau, Lkr. Tuttlingen. Fundberichte. 1990;15: 175–211.

87. Dehn R. Neue Grabfunde der Schnurkeramik und der Glockenbecherkultur von Stetten ad Donau, Krs. Tuttlingen. Archäologische Nachrichten aus Baden. 1991;45: 3–9.

88. Toussaint MP. Excavating Gender: The Embodiment and (Re)presentation of Social Relations in Mierzanowice Communities of the Early Bronze Age. Martin D, editor. PhD, University of Nevada. 2020. Available: https://www.proquest.com/dissertations-theses/excavating-gender-embodiment-re-presentation/docview/2436388249/se-2

89. Dresely V, Müller J. Die absolutchronologische Datierung der Schnurkeramik im Tauber- und im Mittelelbe-Saale-Gebiet. Die absolute Chronologie in Mitteleuropa 3000 - 2000 vChr = The absolute chronology of Central Europe 3000 - 2000 BC. fis.uni-bamberg.de; 2001. pp. 287–318.

90. Липский АН, Вадецкая ЭБ. Могильник Тас Хазаа. Окуневский сборник. 2006;2: 9–52.

91. Allentoft ME, Sikora M, Sjögren K-G, Rasmussen S, Rasmussen M, Stenderup J, et al. Population genomics of Bronze Age Eurasia. Nature. 2015;522: 167–172.

92. Kristiansen K, Allentoft ME, Frei KM, Iversen R, Johannsen NN, Kroonen G, et al. Re-theorising mobility and the formation of culture and language among the Corded Ware Culture in Europe. Antiquity. 2017;91: 334–347.

93. Evans C, Lucy S, Patten R. Riversides: Neolithic Barrows, a Beaker Grave, Iron Age and Anglo-Saxon Burials and Settlement at Trumpington, Cambridge. McDonald Institute for Archaeological Research, University of Cambridge; 2018.

94. Lauermann E. Frühbronzezeitliche Bestattungen im Bereich einer bronzezeitlichen Siedlung in Unterhautzental, Gem. Sierndorf, Niederösterreich. Archaeologia Austriaca. 1991;75: 63–78.

95. Kaiser G. Das frühbronzezeitliche Graberfeld von Unterwölbling. p B St Pölten, NÖ Archaeologia Austriaca. 1962;32: 35–61.

96. Bueno Ramírez P, Barroso R, de Balbín R. 5000 años atrás: Primeros agricultores y metalúrgicos en el Valle de Huecas (Huecas, Toledo). Toledo: Area de Prehistoria Universidad de Alcalá de Henares; 2012.

97. Stenberger M, Dahr E, Munthe H. Das Grabfeld von Västerbjers auf Gotland. Stockholm: Wahlström & Widstrand; 1943.

98. Eriksson G. Part-time farmers or hard-core sealers? Västerbjers studied by means of stable isotope analysis. Journal of Anthropological Archaeology. 2004;23: 135–162.

99. Ковалев АА. Могильник Верхний Аскиз I, курган 2. Окуневский сборник. СПб: Петро-РИФ; 1997. pp. 80–112.

100. Buchvaldek M, Koutecký D. Vikletice: ein schnurkeramisches Gräberfeld. Prague: Universita Karlova; 1970.

101. Nowak M, Musiał-Łaczek B, Włodarczak P. Grave of the Corded Ware culture from Węgrzce, Kraków District. Folia Quat. 2020;88: 63–78.

102. Костылева ЕЛ, Уткин АВ. Нео-энеолитические могильники Верхнего Поволжья и Волго-

Окского междуречья. Москва: Таус; 2010.

103. Langdon PG. Bedford Modern School Museum: Illustrated Guide: Descriptive of the Collection of Bedfordshire Antiquities Now Assembled in the Bedford Modern School Museum. Bedford: Bedfordshire Newspaper Standard Co.; 1925.
